# Interventions Addressing Social Needs in Perinatal Care: A Systematic Review

**DOI:** 10.1089/heq.2020.0051

**Published:** 2021-03-04

**Authors:** Ana M. Reyes, Precious W. Akanyirige, Danielle Wishart, Rabih Dahdouh, Maria R. Young, Araceli Estrada, Carmenisha Ward, Cindy Cruz Alvarez, Molly Beestrum, Melissa A. Simon

**Affiliations:** ^1^ Feinberg School of Medicine, Northwestern University, Chicago, Illinois, USA.; ^2^ Center for Health Equity Transformation, Northwestern University Feinberg School of Medicine, Chicago, Illinois, USA.; ^3^ Loyola University Chicago Stritch School of Medicine, Maywood, Illinois, USA.; ^4^ Galter Health Sciences Library & Learning Center, Northwestern University Feinberg School of Medicine, Chicago, Illinois, USA.

**Keywords:** perinatal care, screening, social determinants, underserved populations, referral

## Abstract

**Introduction:** Health is impacted by a wide range of nonmedical factors, collectively termed the social determinants of health (SDoH). As the mechanisms by which these factors influence wellness and disease continue to be uncovered, health systems are beginning to assess their roles in addressing patient's social needs. This study seeks to identify and analyze clinic-based interventions aimed at addressing patients' social needs in perinatal care, including prenatal, antepartum, and postpartum care.

**Methods:** We conducted a search of six databases through May 2020 for articles describing screening or intervention activities addressing social needs in at least one SDoH domain as defined by Healthy People 2020. We required that studies include pregnant or postpartum women and be based in a clinical setting.

**Results:** Thirty-one publications describing 26 unique studies were identified. Most studies were either randomized-controlled trials (*n*=10) or observational studies (*n*=7) and study settings were both public and private. The mean age of women ranged from 17.4 to 34.1 years. Most studies addressed intimate partner violence (*n*=19). The next most common need addressed was social support (*n*=5), followed by food insecurity (*n*=3), and housing (*n*=2). Types of interventions varied from simple screening to ongoing counseling and case management. There was wide heterogeneity in outcomes investigated. Most IPV interventions that included counseling or ongoing support resulted in reduced IPV recurrence and severity. No intervention with only screening showed a reduction in rate of IPV.

**Conclusion:** This systematic review shines light on several avenues to support pregnant and postpartum women through interventions that embed acknowledgment of social needs and actions addressing these needs into the clinical environment. The results of this review suggest that interventions with counseling or ongoing support may show promise in alleviating social risk factors and improving some clinical outcomes. However, the strength of this evidence is limited by the paucity of studies. More rigorous research is imperative to augment the knowledge of social needs interventions, especially in domains outside of IPV.

## Introduction

Evidence indicates that the social determinants of health (SDoH) shape health in powerful ways.^1,2^ The SDoH have been defined as the conditions in which people are born, grow, live, work, and age.^1^ SDoH include a wide range of social, structural, and economic factors that relate to housing, access to healthy food, transportation, education, crime and violence, and the environment, among other conditions that contribute to a person's quality of life. Fundamental to the study of SDoH is understanding that differences in how population groups experience these conditions are key drivers of disparities in health and health care. Structural racism exacerbates the differential experience of these social and economic conditions, especially among minority communities.^3^

While SDoH refer to social conditions at the level of communities, social needs refer to the acute social and economic challenges of individuals, which are often direct consequences of those broader social conditions.^4^ The health system has long recognized that SDoH are intricately tied to the development of disease and efforts to address SDoH have been described as looking upstream. However, the extent to which the health system can intervene upon SDoH in the setting of clinical care is limited because community-level conditions are often governed by entities outside the health system such as local and national policies. An alternative approach to improving social conditions is addressing the social needs of individual patients, targeting a “middle stream” that is further upstream than traditional medical care, but downstream from the community-level SDoH. Traditionally, addressing individuals' acute social needs has been the role of social service agencies, but a growing number of health systems are beginning to incorporate practice-based interventions for social needs into clinical care.^5,6^ In the primary care literature, some studies have shown that addressing social needs in the clinic increased patients' use of preventive health care.^7,8^

In response to this growing area of practice, the National Academies of Medicine published a consensus report in which they describe activities of health systems that facilitate the integration of social needs care into clinical care.^9^ Using transportation as an example, they suggest that health systems can identify social risks and assets of patients by asking their patients about access to transportation. They also suggest that health systems can reduce social risk by assisting in connecting patients with relevant resources, for example, by providing transportation vouchers so that patients can reliably travel to their health care appointments. These are not novel interventions, but integrating these actions into the clinical care environment is a newer focus of practice.

The idea of unmet social needs as a barrier to accessing health care is of special interest in perinatal care. In perinatal care, visits are frequent and occur in a concentrated period of time; to illustrate, the recommended American Congress of Obstetrics and Gynecology (ACOG) prenatal visit schedule consists of visits every 4 weeks until 28 weeks, every 2 weeks until 36 weeks, and every week until delivery.^10^ The prenatal period is a critical time in pregnancy where essential health screening, pregnancy education, and counseling are performed.^11^ Inadequate prenatal care, defined in one study as fewer than or equal to four antenatal visits, or first visit in the third trimester, has been linked to adverse neonatal outcomes.^12^ Unfortunately, only 77.1% of women initiate pregnancy care in the first trimester.^13^ This number is lower among certain minority groups; only 66.5% of African American and 63.0% of American Indian/Alaska native women receive prenatal care in the first trimester.^13^ These disparities in prenatal care utilization are hypothesized to be linked to social needs, as women have cited lack of transportation, psychosocial barriers, and low health literacy as barriers to care.^14–16^

The objective of this systematic review was to summarize the evidence on interventions that address social needs in perinatal care settings. For the purposes of this review, we use the term “intervention” to describe any clinic-based activity that addresses patients' social needs, including screening, education, counseling, and referral to support services. In this study, we define “perinatal care” as all pregnancy-related medical care, including prenatal, antenatal, and postpartum care.

## Methods

### Data sources

We developed our search strategy in consultation with a medical librarian, which we based on previously published reviews conducted on interventions for social needs in health care settings.^17,18^ Search terms included key words in four domains: (1) pregnant or postpartum women; (2) SDoH; (3) screening and interventions; and (4) outcomes. The complete description of our search strategy is available in the Supplementary Appendix. We searched six databases (PubMed, Embase, Cochrane, CINAHL, Scopus, and Web of Science) for studies describing health care-based interventions published through June 27, 2019, in English. We repeated the search on May 6, 2020, to identify articles published in the time since we began the review. We did not place lower limits on the date range of the search.

### Study selection

Search results were imported into the Rayyan QCRI web app for systematic reviews.^19^ Articles were screened by title and abstract for relevance by a team of reviewers (A.M.R., P.W.A., M.R.Y., R.D., and A.E.). To increase inter-rater reliability, a calibration between reviewers was conducted. The first 100 articles were independently screened by each reviewer and the decision for inclusion was compared. Discrepancies were solved by open discussion between reviewers. The remaining articles were divided evenly among the team and screened by two independent reviewers, with ties broken by a third reviewer.

To be eligible for inclusion, studies had to describe interventions that (1) involved pregnant or postpartum women; (2) addressed a social need in one or more Healthy People 2020 SDoH domains (economic stability, education, social, and community context, health and health care, or neighborhood and built environment);^20^ and (3) took place in whole or in part in a health care setting that we defined as a physical location where women received medical care outside of the home. This included medical care that was given by physicians, physician assistants, nurses, or nurse midwives. Studies describing home visiting, case management, and community health worker programs were only included if the programs were part of an intervention that was clinic based. For example, a study describing an intervention in which a patient is referred to a home visiting program after screening positive for transportation needs during a prenatal appointment would meet the inclusion criteria, while a study describing a home visiting program alone would not.

The Healthy People 2020 framework gives examples of specific social needs in each SDoH domain.^20^ We used these examples to guide study selection but did not limit the selection to only these examples. Both observational and experimental studies were included. When conducting reviewer calibration, we identified areas of misalignment between readers' interpretations of study inclusion. These were settled through group discussion before continuing with study reviews. Although some definitions of SDoH include diet and exercise, substance use, and mental health,^21^ the Healthy People 2020 definition does not.^20^ Thus, we only included studies describing screening/interventions for substance use (including smoking and alcohol use), mental health (depression, post-traumatic stress disorder [PTSD]), and nutrition if they also addressed a Healthy People 2020 domain (economic stability, education, etc.). We included interventions for intimate partner violence (IPV) as IPV is part of crime and violence, which lies under the larger Healthy People 2020 category, neighborhood and the built environment. We did not consider novel methods of prenatal care (e.g., group prenatal care) because we wanted to identify interventions that can be translated to routine clinical care.

We chose to limit studies to those in high-income countries upon recognizing that the research questions could best be answered by evaluating interventions in countries where patients' social needs were most similar to those experienced by U.S. patients. We used World Bank categories to define country income level.^22^

### Data extraction

A standard template was constructed in Microsoft Excel to extract the following data from included studies: year, country or region, study design, setting, participant inclusion/exclusion criteria, population characteristics, social need addressed, description of intervention including who carried out the activities, screening tool used, conceptual framework, and if applicable, whether there was a referral process and/or mention of specific community partners. Studies were assessed for risk of bias and assigned quality ratings based on criteria for randomized-controlled trials (RCTs) and observational studies in the Grading Recommendations Assessment Development and Evaluation (GRADE) approach.^23,24^ Data from each article were extracted independently by two reviewers and discrepancies were discussed between reviewers. Given the heterogeneity of interventions and outcomes across studies, no meta-analysis was performed.

## Results

**FIG. 1.**
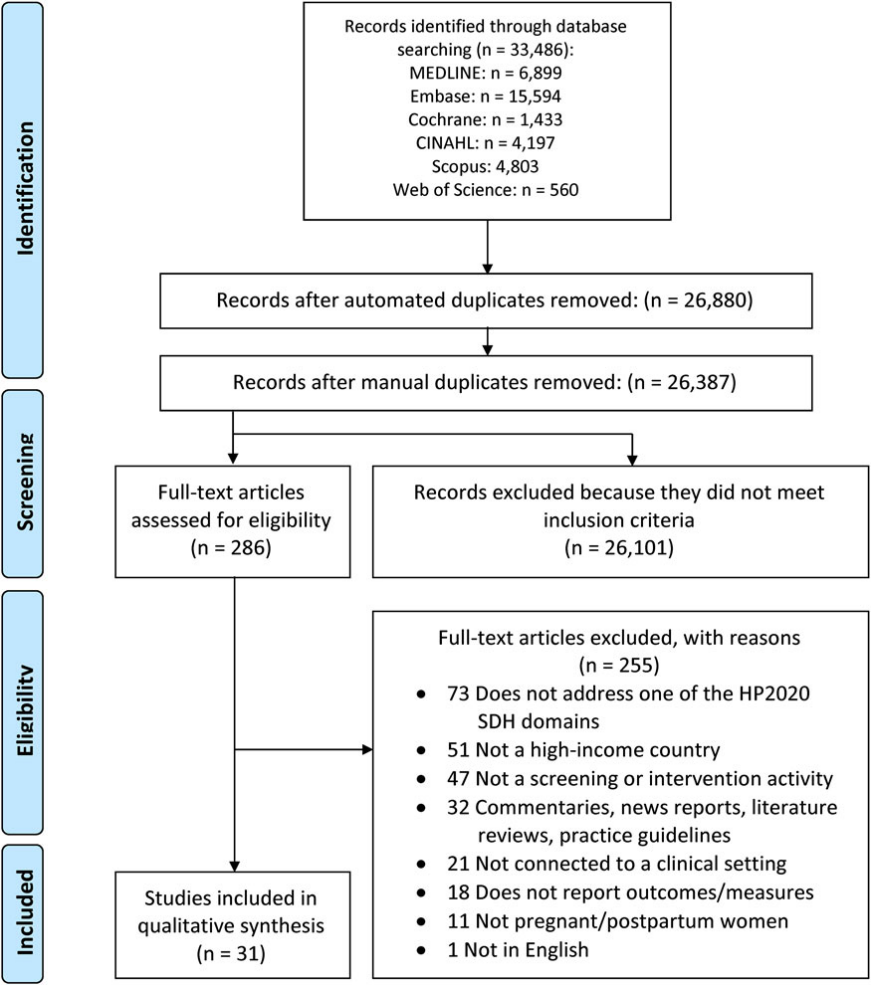
PRISMA 2009 flowchart showing search results through May 6, 2020, and the sequential study selection process. CINAHL, Cumulative Index to Nursing and Allied Health Care; HP2020, Healthy People 2020; SDH, social determinants of health.

Thirty-one articles describing 26 unique studies met all the inclusion criteria (6 articles were based on one study, the District of Columbia Health Outcomes of Pregnancy Education [DC-HOPE] study).^25–30^ The sequential selection process is detailed in Figure 1. Most studies were RCTs (*n*=10)^25–30,31–39^ or observational studies (*n*=7).^40–46^ The remainder of the study designs and quality ratings for all articles are detailed in Table 1.

**Table 1.** Study Characteristics (*n*=31) by Intervention Category; Interventions Addressing Needs in Perinatal Care, High-Income Countries, Through May 2020

Author (year)	Setting	Sample				Control condition	Screening tool (modality)	Social determinants addressed ()^c^	Quality	Study design
		Inclusion	Size^a^	Mean Age^a,b^ (SD)	Race/ethnicity^a^					
**Screening interventions**										
Bernazzani et al. (2005)^40^	United Kingdom (inner-city antenatal clinic)	Project 1: Pregnant, past or current major depressive episode	85	32 (4.7)	Two-thirds Caucasian	N/a	Contextual Assessment of Maternal Experience interview questions (in-person with research staff	Social support	Low	RCT
		Project 2: Pregnant women reporting material deprivation	60	25 (7.4)	39% Caucasian, 33% black Caribbean, 17% black African, 11% mixed	N/a				
Calderón et al. (2008)^32^	San Francisco (five prenatal care clinics)	Pregnant, English-speaking adults reporting IPV	34/27	27 (6.4)/27 (6.6)	10/5 (31%/19%) Hispanic/Latina, 11/9 (34%/33%), black or African American, 5/9 (16%/33%) white, 6/4 (19%/15%) other or multiple races	Usual care	Questions derived from California Adult Tobacco Survey, Abuse Assessment Screen, Mullen et al. (1991)^68^ and Chasnoff at al. (2001)^69^ (computer-based, self-reported with provider “cueing sheet”)	IPV, (alcohol and drug use)	High	RCT
Carroll et al. (2005)^47^	Canada (community prenatal care clinics)	Pregnant between 12 and 30 weeks of gestation, able to read and write in English	98/129	29.1 (5.4)/29.4 (5.2)	Not reported	Usual care	ALPHA form questions (in-person with prenatal care provider at appointment)	IPV, social support, (depression)	Moderate	Cluster-RCT
Chang et al. (2012)^41^	Boston (high-volume hospital with previously established high rate of IPV screening)	Pregnant, English-speaking adults coming for first obstetric visit at the study site	250	25 (5.0)	116 (48%) Caucasian, 115 (47%) African American, 13 (5%) other	N/a	Six-question questionnaire (computer-based, self-administered)	IPV	Low	Observational
Connelly et al. (2000)^42^	California (hospital at time of delivery)	English or Spanish-speaking newly delivered mothers	436	23.4 (6.2)	176 (40%) Hispanic, 100 (23%) African American, 119 (27%) Caucasian, and 41 (9%) Asian, Pacific Islander, Native American/other participants	N/a	Single-item hospital screen vs. 10-item Family Checklist (in-person interview by paraprofessional hospital staff)	IPV	Moderate	Observational
Harrison (2008)^51^	Minneapolis (four FQHCs)	Pregnant women	1356	235 (17.3%) age >17, 247 (18.2%) age 18–19, 474 (35.0%) age 20–24, 222 (16.4%) age 25–29, 178 (13.1%) age 30 or older	606 (44.0%) African American, 264 (19.2%) Asian/Pacific Islander, 217 (15.7%) Hispanic (any race), 192 (13.9%) American Indian, 75 (5.4%) white, 24 (1.7%) bi/multiracial	N/a	Prenatal Risk Overview (in-person by RN or LPN, social worker, MA, or other health care worker and recorded in EHR)	Access to telephone, transportation, IPV, social support, housing instability, food insecurity, legal system involvement (depression, cigarette smoking, alcohol use, illicit drug use)	Very low	Cross-sectional
Price et al. (2019)^50^	Australia (10 public birthing hospitals)	Pregnant women less than 37 weeks gestation with English proficiency	735	27.5 (6.3)	Not reported	N/a	10-item brief risk factor survey (self-administered)	IPV, ETSE, housing quality, social support, high school completion, employment, poverty/income (smoking, alcohol/drug use)	Very low	Cross-sectional
Priest et al. (2008)^44^	Australia (antenatal midwives clinic)	Women attending the clinic	2142	31.2 (1.0)	Not reported	N/a	EPDS and Antenatal Risk Questionnaire with referral process and care plan based on Psychosocial Risk Index score (self-report, referral facilitated by midwives)	IPV, (depression, alcohol/drug use)	Very low	Observational
Zachek et al. (2019)^49^	California (public hospital for predominantly low-income and minority women)	Pregnant adults <20 weeks of gestation and currently working	69		Not reported	N/a	Occupational and environmental exposure questionnaire (self-administered)	Workplace exposures	Very low	Cross-sectional

Author (year)	Setting	Sample				Control condition	Intervention (theoretical framework)	Social determinants addressed ()^c^	Quality	Study design
		Inclusion	Size^a^	Mean age^a,b^ (SD)	Race/ethnicity^a^					
**Brief interventions**										
Gance-Cleveland et al. (2019)^48^	Colorado (midwifery clinic)	Pregnant women, English-speaking attending a return visit at participating clinic	20	Not reported	Diverse backgrounds including African, black, Eastern Indian, white, and Hispanic	N/a	*StartSmart* tablet-based application for support with screening, motivational interviewing, and referral when problems are identified (SBIRT)	IPV, (anxiety, depression, alcohol/drug use, tobacco use, obesity)	Very low	mHealth Development Process
Humphreys et al. (2011)^34^	San Francisco (five prenatal care clinics)	Pregnant, English-speaking adults reporting IPV	25/25	29.1 (6.8)/26.4 (7.3)	9/9 (32%/36%) Hispanic/Latino, 7/4 (28%/16%) black or African American, 5/10 (20%/40%) white, 5/2 (20%/8%) other or multiple races	Usual care	Computer-based self-reported screening, 15-min Video Doctor assessment, and counseling, with cueing sheet given to provider before patient is seen (motivational interviewing)	IPV, (tobacco use, alcohol/drug use)	Moderate	RCT
Price et al. (2017)^46^	Virginia (clinics in two rural and two urban communities qualified as federal health shortage areas)	Pregnant patients eligible for the Maternal, Infant, and Early Childhood Home Visiting Program of the Virginia Department of Public Health	1515	Not reported	At urban sites: equally Caucasian and African American; at rural sites: 78% Caucasian	N/a	*Behavioral Health Integrated Centralized Intake:* supportive screening, brief intervention, and referral to treatment or community intervention by paraprofessionals at first point of eligibility determination for maternal and child health home visiting (transtheoretical model, SBIRT)	IPV, (alcohol/drug use, tobacco)	Low	Observational
Sayakhot et al. (2016)^36^	Australia (maternity diabetes clinic at Western Health hospital)	Singleton pregnancy women age 18–45 years, newly diagnosed with GDM, able to read/write in English, without preexisting diabetes	56/60	Not reported	Not reported	Standard clinic-based 1.5-h GDM education class	Web-based education intervention aimed at a low level of literacy in addition to standard clinic-based 1.5-h group education class	Health literacy	High	RCT
Van Parys et al. (2017)^38^	Belgium (11 hospitals)	Pregnant adults able to fill out a questionnaire in Dutch, French, or English	115/108	27.9 (5.0)/27.7 (5.4)	Not reported	Questionnaire for IPV and “thank you” card	Questionnaire for IPV and referral card with contact details of IPV services and tips to increase safety behavior	IPV	High	RCT
**Counseling interventions**										
Backonja et al. (2016)^31^	Washington, DC (six prenatal care clinics serving primarily minority women)	Pregnant women ≤28 weeks gestation, English speaking, self-identifying as African American or Latina, and reporting any of four risks (active smoking, ETSE, depression, IPV)	521/523	23 (5.49)/24 (5.32)	African American	Usual care	Eight sessions of reproductive health education and tailored counseling to address psychosocial and behavioral risks, delivered within prenatal care clinic by Master's-level social worker or psychologist (social cognitive theory, transtheoretical model, CBT, Dutton's empowerment model)	Health literacy	High	RCT
El-Mohandes et al. (2008)^25,d^	Washington, DC (six prenatal care clinics serving primarily minority women)	Pregnant women ≤28 weeks gestation, English speaking, self-identifying as African American, and reporting ETSE	452/461	24.4 (0.3)/24.8 (0.3)	African American	Usual care	Four to eight sessions of clinic-based tailored counseling issued by Master's level social worker or psychologist during prenatal care appointment based on result of 4-risk self-administered screen (social cognitive theory, transtheoretical model, CBT, Dutton's empowerment model)	IPV, ETSE, (depression, smoking)	High	RCT
El-Mohandes et al. (2010)^26,d^	Washington, DC (six prenatal care clinics serving primarily minority women)	Pregnant women ≤28 weeks of gestation, English-speaking, self-identifying as African American or Latina and reporting any of four risks (active smoking, ETSE, depression, IPV)	335/356	23.4 (4.8)/24.1 (5.1)	African American	Usual care	See El-Mohandes (2008)^25^	IPV, ETSE, (depression, smoking)	High	RCT
El-Mohandes et al. (2011)^27,d^	Washington, DC (six prenatal care clinics serving primarily minority women)	See El-Mohandes (2010)^26^	403/416	189/165 (47.0%/39.8%) age 18–22, 118/136 (29.4%/32.8%) age 23–27, 95/114 (23.6%/27.5%) age >=28	African American	Usual care	See El-Mohandes (2008)^25^	IPV, ETSE, (depression, smoking)	High	RCT
Katz et al. (2008)^30^	Washington, DC (six prenatal care clinics serving primarily minority women)	See El-Mohandes (2010)^26^	521/523	24.4 (5.5)/24.8 (5.3)	African American	Usual care	See El-Mohandes (2008)^25^	IPV, ETSE, (depression, smoking)	High	RCT
Kiely et al. (2010)^29,d^	Washington, DC (six prenatal care clinics serving primarily minority women)	See El-Mohandes (2010)^26^	169/167	24.5 (5.8)/24.5 (5.4)	African American	Usual care	See El-Mohandes (2008)^25^	IPV, ETSE, (depression, smoking)	High	RCT
Kiely et al. (2013)^28,d^	Washington, DC (six prenatal care clinics serving primarily minority women)	See El-Mohandes (2010)^26^	256 reporting new risks after baseline, 594 reporting no new risks after baseline	24.1 (5.1) in group reporting new risks after baseline, 24.5 (5.4) in group reporting no new risks after baseline	African American	Usual care	See El-Mohandes (2008)^25^	IPV, ETSE, (depression, smoking)	Moderate	RCT
Parker et al. (1999)^55^	Texas and Virginia (public clinics that offer a variety of health services for women and children)	Women receiving prenatal care and reporting abuse who considered themselves still in a relationship	132/67	Not reported	Not reported	Routine prenatal care	Three sessions of one-on-one empowerment counseling offered in English and Spanish (empowerment model)	IPV	Very low	Quasi-experimental
Tiwari et al. (2005)^37^	Hong Kong (public hospital)	Pregnant women between 18 and 30 weeks of gestation	55/55	30 (5.1)/31 (5.3)	Not reported	Standard care and a wallet size card with information on community resources for abused women	Abuse Assessment Screen plus 30-min empowerment counseling by Master's level plus brochure reinforcing information provided midwife (Parker's empowerment model, Roger's client-centered therapy)	IPV	High	RCT
Zlotnick et al. (2011)^39^	Rhode Island (two primary care clinics and one private OBGYN clinic)	Pregnant adults between 18 and 40 years old who disclosed IPV at recruitment	28/26	24.2 (4.4)/23.5 (4.7)	35.7%/42.3% white, 42.9%/42.3% Hispanic, 14.3%/7.7% black, 7.1%/7.7% other/multiracial	Usual care and a listing of resources for IPV	Four 60-min individual counseling sessions delivered over a 4-week period during pregnancy and one “booster” session at 2 weeks postpartum (interpersonal psychotherapy)	IPV	High	RCT
**Interventions with ongoing support services**										
Cohen et al. (2011)^53^	United Kingdom (midwifery clinic for teenage mothers)	Pregnant teenagers presenting for prenatal care at the clinic	65/65	17.4 (1.4)/17.6 (0.9)	Not reported	Usual midwifery care provided by the clinic	Usual midwifery care provided by the clinic plus additional psychosocial support	Social support	Moderate	Pretest/post-test
Curry et al. (2006)^33^	Health Maintenance Organization in the Pacific Northwest^g^ and rural university clinic in the Midwest^h^ (prenatal care clinics)	Pregnant women 13 to 23 weeks of gestation who spoke English and identified as at-risk for abuse	499/501^e^	29.71 (5.91)/27.73 (5.87)^e^	338/410 (67.6%/82.0%) Caucasian, 81/61 (16.2%/12.2%) African American, 22/7 (4.4%/1.4%) Hispanic, 19/13 (3.8%/2.6%) Asian/Pacific Islander, 6/0 (1.2%/0%) Native American, 34/9 (6.8%/1.8%) other^e^	Usual care plus IPV safety card with phone numbers for local and national resources	*Faces of Abuse* video, access to Connections NCM 24/7 and individualized nursing case management throughout pregnancy based on self-identified needs plus IPV safety card with phone numbers for local and national resources (Landenburger's theory of entrapment)	IPV	Moderate	RCT
Kramer et al. (2012)^43^	Wisconsin (prenatal clinics, inpatient units, emergency departments, private OB offices)	Pregnant and newly delivered women who screened positive for IPV and were referred to the Safe Moms Safe Babies program	418	Not reported	Not reported	N/a	Enrollment into Safe Moms Safe Babies nurse case management during health care visit (empowerment model)	IPV, (alcohol/drug use)	Very low	Observational
McFarlane et al. (2000)^35^	Large city in the Southwest United States (two prenatal clinics of the health department serving 97% Hispanic patients)	Pregnant patients attending the clinic for first prenatal appointment who screened positive for abuse	329	23.7 (5.5)	Not reported	Three interventions compared		IPV	Low	RCT
						Brief intervention—wallet size card with phone numbers of local agencies and safety planning information				
			98	24.2 (5.4)		Counseling—unlimited access to counseling services of a female, bilingual Spanish-speaking counselor, physically located in the clinic and available when the clinic was open from intake until delivery				
			118	23.8 (5.4)		Outreach—same unlimited access to counseling services plus a “mentor mother” who offered support, education, referral, and assistance in accessing community resources through personal visits and phone calls from intake until delivery				
Taft et al. (2015)^54^	Australia (community-based MCH teams)	Postpartum women	1269/1352	34.1 (4.5)/34.0 (4.6)	Not reported	Usual care including government-mandated face-to-face domestic violence screening at 4 weeks postpartum	A self-completion maternal health and well-being checklist and enhanced screening with nurse mentors (in-person by nurses)	IPV	High	Cluster-RCT
Trapl et al. (2017)^45^	Ohio (one FQHC, two WIC sites, one community health center in neighborhoods with limited access to traditional food retailers and healthy foods)	Pregnant women less than 24 weeks of gestation	75	23.8 (5.1)	51 (68.9%) black, 10 (13.5%) white, 13 (17.6%) Hispanic	N/a	Monthly nutritional counseling and $40 vouchers to local farmer's market, at usual prenatal care visits for 4 months (theory of implementation intentions and repeated behavior)	Food insecurity, access to healthy foods	Very low	Observational
Watt et al. (2015)^52^	Southwest United States (primary care clinic serving low-income, Hispanic women)	Pregnant, Spanish-speaking adults receiving prenatal care at the clinic in their first trimester	32/29	28.7/26.7	Not reported	Usual care	Vouchers for fruits and vegetables from farmer's markets, 1.5-h nutrition classes/cooking classes conducted in Spanish, lactation counseling (biopsychosocial framework)	Food instability, access to healthy foods, health literacy	Very low	Formatting

^a^ Numbers are reported as intervention/control unless otherwise noted.

^b^ Age is reported in years.

^c^ Social determinants in parentheses are included in some definitions of social determinants of health, but not the Healthy People 2020 definition.

^d^ El-Mohandes (2008),^25^ El-Mohandes (2010),^26^ El-Mohandes (2011),^27^ Kiely et al. (2013),^28^ Kiely (2010),^29^ and Katz (2008)^30^ are all part of the same larger study.

^e^ Sample size, mean age, and race/ethnicity data for Curry et al. are reported as [Pacific Northwest clinic/Rural Midwest clinic].

ALPHA, Antenatal Psychosocial Health Assessment; DC, District of Columbia; EHR, electronic health record; EPDS, Edinburgh Postnatal Depression Scale; ETSE, environmental tobacco smoke exposure; FQHC, federally qualified health center; GDM, gestational diabetes mellitus; IPV, intimate partner violence; LPN, licensed practical nurse; MA, medical assistant; MCH, maternal child health; N/a, not applicable; NCM, nurse case management; PTSD, post-traumatic stress disorder; RCT, randomized-controlled trial; RN, registered nurse; SBIRT, screening, brief intervention and referral to treatment; WIC, Women, Infants and Children.

### Settings

Most interventions took place in prenatal care clinics (*n*=15),^25–30,31–34,39,40,43,47,48^ hospitals (*n*=8),^36–38,41–43,49,50^ or primary care clinics (*n*=5).^39,45,51,52^ Some took place in midwife clinics (*n*=3).^44,53,54^ Settings were both private and public and included private obstetric offices (*n*=2),^39,43^ public clinics and hospitals (*n*=4),^35,49,50,55^ federally qualified health centers (*n*=3),^45,46,51^ Women, Infant, and Children (WIC) sites (*n*=1),^45^ academic settings (*n*=7),^25–30,49^ and community settings (*n*=4).^43,45,46,54^ Studies were conducted in the United States (*n*=17),^25–30,31–35,39,41–43,45,46,48,49,51,52,55^ Australia (*n*=4),^36,44,50,54^ the United Kingdom (*n*=2),^40,53^ Canada (*n*=1),^47^ Hong Kong (*n*=1),^37^ and Belgium (*n*=1).^38^

### Populations

Seven interventions targeted specific patient populations: teenagers,^53^ women with gestational diabetes,^36^ Hispanic women,^35^ low-income Spanish-speaking women,^51^ African American women,^25–31^ and low-income minority women.^7^ Black or African American women comprised more than 20% of the sample in nine studies^25–32,34,40–42,45,46,51^ and Hispanic women comprised more than 15% of the sample in five studies.^36,39,42,45,51^ Native women were included in three studies but represented less than 15% of the sample in these studies.^33,42,51^ Notably, most studies outside the United States did not report race/ethnicity data.^36–38,44,47,50,53,54^ The mean age of women ranged from 17.4 to 34.1 years.

### Social needs

**FIG. 2.**
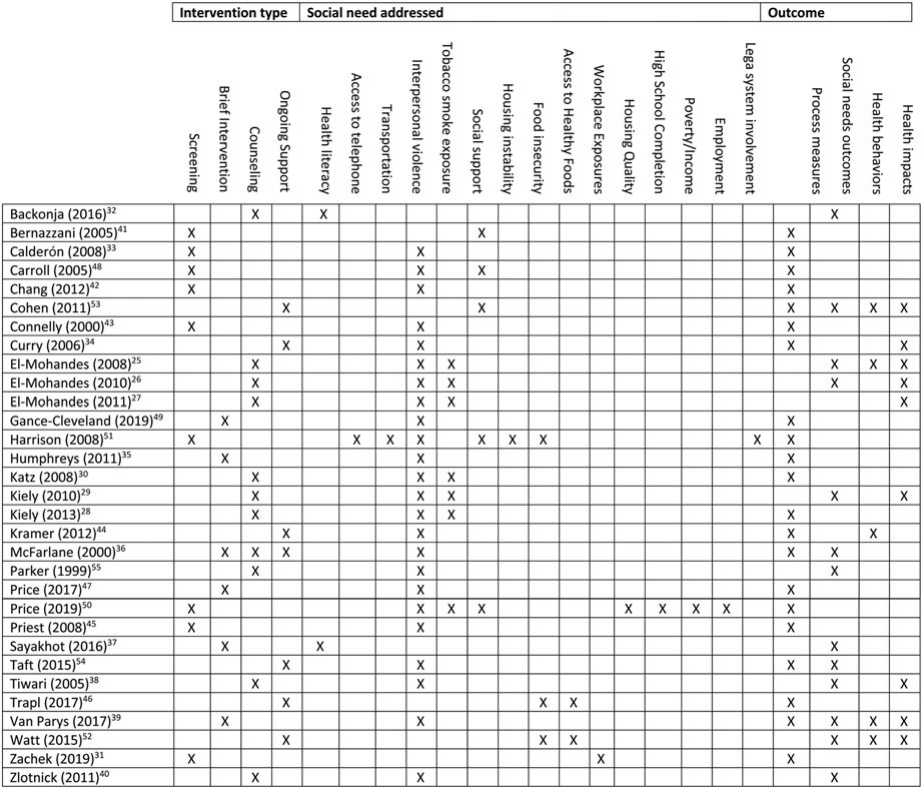
Interventions by type, social needs category, and outcome.

Figure 2 provides a visual display of the range of social needs addressed by the interventions in this review. Overall, 20 interventions addressed a single social need and six interventions addressed multiple needs. The vast majority of interventions addressed IPV (*n*=19).^25–30,32–35,37–39,41–44,46–48,50,51,54,55^ Of these, 15 interventions solely addressed IPV, while the other four also addressed additional social needs. The Antenatal Psychosocial Health Assessment form used by Carroll et al. asked patients about social support in addition to IPV, and the DC-HOPE studies addressed both tobacco smoke exposure and IPV. The other two interventions that addressed IPV did so in the context of multi-item screening surveys for a broad range of social needs. In the study by Price et al., a 10-item brief risk factor survey asked patients about IPV, environmental tobacco smoke exposure (ETSE), housing quality, high school completion, employment, and poverty/income.^50^ In the Prenatal Risk Overview by Harrison et al., patients were asked about access to a telephone, transportation, IPV, social support, housing instability, legal system involvement, and food insecurity.^51^

Food security and access to healthy foods were addressed in three studies.^45,51,52^ Expanding/Building on simple screening in Harrison et al., the interventions in Trapl et al. and Watt et al. provided patients with vouchers to local farmer's markets.

Housing stability and quality were addressed in only two studies, the same multi-item screening surveys as mentioned above.^50,51^ Education, employment, and poverty were only addressed in the 10-item brief risk factor survey by Price et al.^50^ It should be noted that no intervention went beyond screening for housing, education, employment, or poverty. One study screened for workplace exposures.^49^

Two studies addressed health literacy: Sayakhot et al. offered a web-based education module for gestational diabetes that was issued during clinic visits and Backonja et al. incorporated reproductive health education into regular clinic-based counseling sessions.^31,36^

No studies addressed civic participation, discrimination, or incarceration history, which fall under the social and community context domain of Healthy People 2020.

### Interventions and outcomes

To facilitate the reporting of results, we classified interventions into four broad categories: screening (*n*=11), brief (*n*=6), counseling (*n*=6), and ongoing support (*n*=6). One study compared three different interventions, so we cross-counted this intervention in three categories.^35^ We also classified outcomes into four categories: process measures, social needs outcomes, health behaviors, and health impacts, which we based on previous work by Gottlieb et al.^17^ Figure 2 provides a visual display of interventions according to type of intervention, social need(s) addressed, and outcomes reported. Table 2 provides a more detailed breakdown of study outcomes and how they were classified.

**Table 2.** Study Outcomes (*n*=31); Interventions Addressing Social Needs in Perinatal Care, High-Income Countries, Through May 2020

Process measures	Measures of social risks	Health behaviors	Health outcomes
• Validity of screening tool^40,42,49,50,51^ • Risk identification^28,32,34,41,42^ • Acceptability^30,32,33,41,44,46,47^ • Patient-rated helpfulness^32,34,38,43,45,48^ • Length of administration^30,45,49,51^ • Referral/patient use of referrals^33,35,45,46,53,54^ • Screening rate^54^ • Appointment adherence^45^	• Health knowledge^31,36^ • Social support^52,53^ • ETSE^25,26^ • IPV (episodes of violence, severity, behaviors)^29,11,35,37–39,55^	• Physical activity^52,53^ • Healthy diet^52^ • Substance use (smoking, alcohol use)^25,52,53^ • Help-seeking behavior^38,43^ • Safety planning^54^	• Infant birthweight^26,27,29^ • Gestational age at birth^26,27,29^ • Miscarriage, perinatal death, nonlive birth, neonatal hospitalization days^26^ • Maternal weight gain^52^ • Infant development^52^ • Mental health status (depression, PTSD, stress, self-esteem)^25,33,38,52,53^ • Pain and physical functioning^37^ • Breastfeeding^52^

ETSE, environmental tobacco smoke exposure; IPV, intimate partner violence.

The studies in our review were heterogeneous in terms of intervention type, social need(s) addressed, and outcomes reported. Thus, we did not perform a meta-analysis or attempt to make formalized comparisons across studies. Instead, in this narrative section, we summarize the results by type of intervention and outcome, which we report fully in Tables 1 and 2. Study quality based on GRADE criteria is also reported in Table 1. In some studies, a specific referral process to outside organizations was outlined as part of the intervention. Other interventions were carried out in partnership with community organizations. We describe these fully in Table 3.

**Table 3.** Referrals and Community Partnerships Reported in Review Studies (*n*=13); Interventions Addressing Social Needs in Perinatal Care, High-Income Countries, Through May 2020

Study (year)	Social determinants addressed	Health professional referring	Description of referral (community partnership)
Cohen et al. (2011)^53^	Social support	Project midwives	Referrals to specialist services and agencies—benefits agency, local authority housing department, smoking cessation counseling, infant feed counselor, family planning association, condom card scheme, further education college, social services, drug support teams. Part of midwife intervention with additional social support.
Curry et al. (2006)^33^	IPV	Nurse case managers	Referrals given through NCM program. Referrals most commonly for housing and food resources, educational programs, and domestic violence services
Gance-Cleveland et al. (2019)^48^	IPV (anxiety, depression, alcohol/drug use, tobacco use, obesity)	Clinician with support of *StartSmart* mHealth tool	Referral algorithm. If low or no risk, positive behaviors reinforced; if moderate risk, brief intervention such as education handout; if high risk, referral to specialty care
Harrison (2008)^51^	Access to telephone, transportation, IPV, social support, housing instability, food insecurity	RNs and case managers	Referral algorithm. If moderate risk, education or social support offered; If high risk, referral to specialized services such as mental health, drug use assessment, or domestic abuse program
Kramer et al. (2012)^43^	IPV (alcohol/drug use)	County public health nurses case managers	Referrals are made from health care settings into the nurse-led Safe Mom, Safe Baby case management program. Once enrolled, nurse case managers give referrals to needed services. (Partnership between the University of Wisconsin School of Medicine and Public Health and community-based domestic violence partners.)
McFarlane et al. (2000)^35^	IPV	Bilingual counselor with expertise in domestic violence	Women in referral card intervention received referral card with contact information of community services. Women in counseling intervention were referred to domestic violence services by the counselor
Parker et al. (1999)^55^	IPV	Master's or doctorate level nurses	Referrals listed on a brochure, (women were encouraged to attend counseling sessions taught by workers at a local women's shelter)
Price et al. (2017)^46^	IPV	Social workers and paraprofessional outreach workers	Referral algorithm. Triaged assessment with referral protocols based on identified risks. Referral to home visiting support and specialized mental health services
Priest et al. (2008)^44^	IPV	Midwives	Referral triage system at first prenatal visit with subsequent creation of a psychosocial care plan. Based on risk scores, women are offered urgent or routine referral for formal mental health evaluation or additional psychosocial care including early intervention, support, hospital-based treatment programs, community-based resources and services, antenatal group programs, and stress management programs
Taft et al. (2015)^54^	IPV	MCH nurse	Referral to domestic violence services
Trapl et al. (2017)^45^	Food insecurity, access to healthy foods	Health care providers	(Local farmer's markets in Cuyahoga County, Ohio)
Watt et al. (2015)^52^	Food insecurity, access to healthy foods	Physicians	(Local farmer's market and nutrition/cooking classes offered by a local nonprofit community partner)
Zachek et al. (2019)^49^	Workplace exposures	Qualified program staff	Staff reviewed screening results and referred participants to the UCSF occupational and environmental medicine clinic for additional follow-up if necessary

UCSF, University of California San Francisco; NCM, nurse case manager; RN, registered nurse; MCH, maternal child health.

Nine studies in this review were categorized as screening interventions. Four of these studies addressed solely IPV, one addressed IPV and social support, one addressed solely social support, and one addressed workplace exposures. The interventions in Harrison et al. and Price et al., the multi-need screening interventions mentioned above, were also categorized in this group. In terms of outcomes, most studies reported process measures such as patient-reported helpfulness, ease of use, or internal validation of the screening tool. Price et al. compared the results of their 10-item brief risk factor survey that they administered in the waiting room to private interviews and found that their screener concurred with the private interviews in identifying social needs. Zachek et al. also issued their screening tool as a waiting room survey and found that closed-ended questions identified more workplace exposures than open-ended questions did. Patients who screened positive for workplace exposures were then referred to an occupational and environmental medicine clinic for follow-up. The only other screening intervention that utilized a referral process was that in Harrison et al., which used an algorithmic approach with a risk score to refer patients to specialized services based on their reported risk severity. No screening studies reported health outcomes, health behavior outcomes, or measures of social risks. Overall, patients found screening interventions acceptable, helpful, and felt comfortable discussing social needs with clinicians.^32,41,47^

We defined brief interventions as screening with brief education or advice related to the social needs disclosed. We considered the web-based gestational diabetes education tool to be a brief intervention for health literacy.^36^ Of the six studies describing brief interventions, five addressed IPV and one addressed health literacy. The brief interventions for IPV varied in approach and outcomes. One intervention consisted of a tablet-based screening tool with motivational interviewing prompts that were individually generated based on reported risks.^48^ Women interviewed in this study reported feeling that they could better adhere to the treatment guidelines for IPV after using the tablet-based screener. Another intervention was a computer-based screener with a 15-min informational video and prompting card that was given to the provider.^34^ Women in this intervention group had significantly more discussions about IPV with their provider compared with women receiving usual care. The intervention in Price et al. utilized a screening intake form plus brief IPV information and guided referral.^46^ Uptake of referral in this study was low and most women reported being uninterested in specialized services. The investigators found the biggest predictor of successful referral to be women's level of engagement during screening. Two studies addressed IPV using a screening questionnaire combined if a referral card with resources and safety tips. Results showed that the referral cards did not decrease violence scores, IPV victimization, or symptoms of depression, and did not improve help seeking behaviors.^35,38^ The web-based health literacy intervention did improve women's knowledge of gestational diabetes topics compared with women in the control group.^36^

Counseling interventions consisted of interventions where one or more counseling sessions were provided in concert with clinical prenatal care. Six studies on counseling interventions were reported in 11 publications. All counseling interventions addressed IPV, with one also addressing health literacy and one also addressing tobacco smoke exposure.^31^ Six publications reported on the DC-HOPE study, which was an RCT that tested a screening and counseling intervention for IPV, ETSE, depression, and smoking. Screening took place at the patients' first prenatal care appointment and counseling took place over four to eight prenatal care visits. Counseling sessions were individually tailored to patients' reported risks. The studies reported a wide range of outcomes, including reduction in ETSE, recurrent episodes of IPV victimization, cigarette smoking, infant birthweight, gestational age at birth, miscarriage, and symptoms of maternal depression. Women were recruited from six prenatal care clinics serving primarily minority women and all women included in the study identified as black or African American. The study found that the intervention group had significantly fewer very preterm births (<34 weeks) (odds ratio [OR]=0.22, 95% confidence interval [CI]=0.07–0.68) and very low birthweight babies (<1500 g) (OR=0.11, 95% CI=0.01–0.86) compared with control.^26,27,29^ There were no significant differences in preterm births (<37 weeks), low birthweight (<2500 g), miscarriage, perinatal death, nonlive birth, or neonatal hospitalization days. Among women initially reporting ETSE, women in the intervention group were significantly less likely to report ETSE before delivery when controlling for other covariates, including other psychobehavioral risks (OR=0.50, 95% CI=0.35–0.71).^26^ Among women initially reporting IPV, intervention group women were less likely to have recurrent episodes of IPV victimization (OR=0.48, 95% CI=0.29–0.80).^29^ The study by Backonja et al. was adjunct to the DC-HOPE study and incorporated reproductive health education into four to eight counseling sessions. Women receiving the health education scored higher on reproductive knowledge assessments compared with women receiving usual care. Aside from the DC-HOPE studies, there were three other counseling interventions for IPV.^37,39,55^ All consisted of empowerment counseling sessions during the medical visit and all except for the intervention in Zlotnick et al. showed a significant reduction in measures of violence for women in the intervention groups.^35,37,39,55^ While the intervention in Zlotnick et al. did not show a significant reduction in IPV, they did find a moderate reduction in symptoms of depression and PTSD during pregnancy.

Interventions that provided women with regular support outside of the clinic were categorized as interventions with ongoing support. There were seven of these interventions in total, with four for IPV,^33,35,43,54^ one for social support,^53^ and two for food insecurity.^45,52^ The interventions in Curry et al. and Kramer et al. connected women with nurse case management services, which they had access to throughout their pregnancy. These studies had favorable outcomes; intervention group women in Curry et al. showed significantly fewer symptoms of stress at the completion of the program and intervention group women in Kramer et al. performed more safety behaviors to protect against IPV than at baseline. Similarly, the interventions in McFarlane et al. and Taft et al. connected women with nurse mentors who were available to women from the initial visit until delivery. These studies also showed favorable outcomes; intervention group women in McFarlane et al. reported decreased severity of IPV at 2 months postdelivery and intervention group women in Taft et al. showed a significant increase in safety planning. The study by Cohen et al. from the United Kingdom was unique in that it addressed social support amongst pregnant teenagers. In this study, pregnant teenagers were given the support of a nurse midwife who assisted them in connecting with various community services. While results showed that teenagers in the intervention group had increased uptake of certain resources, there were no differences in measures of social support, self-esteem, physical activity, or smoking behavior at 4 weeks postpartum. There were also no differences in uptake of National Health Service services between the intervention and control groups.

We grouped the food insecurity interventions with ongoing support interventions; however, they were structured differently than the interventions for IPV and social support. The interventions in both Trapl et al. and Watt et al. consisted of nutritional counseling given at regular clinic visits. They both partnered with local farmer's markets to issue food vouchers, which were also distributed at regular clinic visits. The intervention in Watt et al. also included a 1.5-h nutrition and cooking class that was taught in Spanish and lactation counseling that was offered at the clinic and over the phone. The interventions in Trapl et al. and Watt et al. did not include ongoing mentoring or case management. Trapl et al. reported appointment adherence and patient use of referrals. Providers felt that the intervention increased appointment adherence, and objectively, 43% of patients kept 100% of appointments and another 31% of patients kept 50–99% of appointments. Using redemption logs from the farmer's market, the investigators found that 56% of participants redeemed at least one voucher. Watt et al. reported a wider range of outcomes. Women in the intervention group had significant improvements in diet, exercise, and depression (*p*≤0.05) compared with women receiving usual care. In addition, women were more likely to breastfeed (*p*=0.07) and their infants were more likely to pass the ages and stages developmental screen (*p*=0.06) than women in the usual care group.

## Discussion

The 26 studies summarized in this review provide an overview of all clinic-based social needs interventions for pregnant and postpartum women that have been reported in the literature to date. They also provide a summary of the evidence for such interventions in improving maternal and infant health outcomes and mitigating social risks and behaviors that have been associated with poor health outcomes.

We choose to discuss interventions for IPV separate from those addressing other social needs domains as IPV interventions represented the majority of interventions identified in our review.

Among interventions addressing IPV, the evidence in our review points to counseling interventions and interventions with ongoing support as the most effective interventions for improving health and health-related outcomes. Overall, most of these studies found significant reductions in recurrent IPV^28,55^ and severity of IPV^35^ or significant improvements in healthy behaviors such as safety planning in Kramer et al. Two studies reported objective health outcomes; the DC-HOPE studies showed a significant reduction in very preterm births and very low birthweight infants and Zlotnick et al. demonstrated a moderate effect of their intervention in reducing symptoms of depression during pregnancy. On the contrary, no screening or brief intervention for IPV showed a reduction in IPV rates and evidence was mixed for increasing safety planning rates.^34,37,54^ These findings are consistent with those of the US Preventive Service Task Force (USPSTF),^56^ which concluded that interventions providing women with ongoing support have moderate benefit, but brief interventions in the absence of ongoing services are not shown to have benefit. Of note, in both our review and that of the USPSTF, the DC-HOPE study was the only study reporting birth outcomes, illustrating the paucity of evidence for such interventions.

Interventions addressing social needs other than IPV were few. Among these, the type of intervention did not correspond to effectiveness of intervention as seen among interventions for IPV. For example, both brief and counseling interventions for health literacy were effective at improving health knowledge.^31,36^ Meanwhile, an ongoing support intervention for social support did not result in women reporting increased social support.^53^ Finally, the counseling intervention for ETSE and the ongoing support interventions for food insecurity resulted in a reduction in tobacco smoke exposure in postpartum women and higher rates of appropriate development among infants, respectively.^25,52^ These results suggest that different social needs may be best addressed by different types of interventions, unique to the need being addressed. Overall, the evidence for social needs interventions in perinatal care beyond those for IPV is lacking and more research is needed to understand the effectiveness of such interventions.

We were surprised by how few interventions for food insecurity were identified in our review given that federal food assistance programs such as the Special Supplemental Nutrition Program for WIC are widely used by pregnant women, with nearly 4 in 10 expectant mothers enrolled in the program.^57^ Numerous studies have concluded that participation in WIC is associated with improved birth outcomes such as higher birthweight, lower likelihood of neonatal intensive care unit admission, lower likelihood of preterm birth, and lower odds of 1-year infant mortality.^58–62^ While several studies mention referring women to food assistance programs, only one specifically mentions WIC. Designing interventions that facilitate enrollment in WIC and implementing them in clinical care settings may be one approach to addressing food insecurity among pregnant and postpartum women, particularly those who are low income. Given that there are numerous governmental and community programs that address other social needs such as transportation and housing, designing interventions that leverage these existing programs might facilitate the study of clinic-based social needs interventions in other domains.

It is worth noting that no study in our review addressed refugee status or racism. Several studies in the immigrant health literature have shown that Asian and African refugees experience higher rates of pregnancy complications compared with women in the local population.^63,64^ Although not unique to immigrant health, the literature discusses how immigrant health is not negatively impacted by one or two factors, but instead by a multitude of inequities, including dealing with day-to-day discrimination and racism.

Our review has several limitations. First, the scope was broad and captured a range of studies describing heterogenous interventions and outcomes, which prevented comparisons across studies. However, this breadth was intentional and necessary to examine such a broad subject matter. Second, while populations were diverse, the highest quality studies in this review were in African American populations and results may not generalize to other populations. In addition, very few Native American/indigenous women were included in the studies in our review, which is concerning given that indigenous women experience similarly elevated maternal health disparities.^16,65^ Our review also does not account for nontraditional care settings or alternative models of prenatal care. These alternative models may be of benefit to women who desire to carry out their pregnancies outside the traditional health care system, for example, on ancestral lands, which often have less access to specialty care.^66^ Finally, we limited our review to peer-reviewed publications and health care settings in high-income countries. We may have excluded gray literature that could have implications for this rapidly growing area of research.

## Conclusion

This review provides an overview of all published interventions for social needs in perinatal care that have been reported in the literature to date. The results of this review suggest that most interventions have been for IPV, and among these, interventions with counseling or ongoing support show the most promise in alleviating social risk factors and improving some clinical outcomes. Few interventions address social needs other than IPV. More rigorous research should be conducted that expands beyond IPV to other social needs domains. We find it worth noting that while some social needs interventions may be beneficial, they do not represent long-term solutions to the underlying social conditions that are the root of many health disparities, including those in maternal and child health.

## Supplementary Material

Supplemental data

## Abbreviations Used

ACOG: American Congress of Obstetrics and Gynecology
ALPHA: Antenatal Psychosocial Health Assessment
CI: confidence interval
DC: District of Columbia
DC-HOPE: District of Columbia Health Outcomes of Pregnancy Education
EHR: electronic health record
EPDS: Edinburgh Postnatal Depression Scale
ETSE: environmental tobacco smoke exposure
FQHC: federally qualified health center
GDM: gestational diabetes mellitus
GRADE: Grading Recommendations Assessment Development and Evaluation
IPV: intimate partner violence
LPN: licensed practical nurse
MA: medical assistant
MCH: maternal child health
N/a: not applicable
NCM: nurse case management
OR: odds ratio
PTSD: post-traumatic stress disorder
RCT: randomized-controlled trial
RN: registered nurse
SBIRT: screening, brief intervention and referral to treatment
SDoH: social determinants of health
UCSF: University of California San Francisco
USPSTF: US Preventive Service Task Force
WIC: Women, Infant, and Children

## References

[1] Blas E, Kurup AS, World Health Organization, eds. Equity, Social Determinants, and Public Health Programmes. Geneva: World Health Organization, 2010

[2] Centers for Disease Control and Prevention. Social Determinants of Health. 2019. Available at https://www.cdc.gov/nchhstp/socialdeterminants/faq.html Accessed 61, 2020

[3] Gee GC, Ford CL. Structural racism and health inequities: old issues, new directions. Du Bois Rev. 2011;8:115–132 25632292 10.1017/S1742058X11000130 PMC4306458

[4] When Talking About Social Determinants, Precision Matters | Health Affairs. Available at https://www.healthaffairs.org/do/10.1377/hblog20191025.776011/full/ Accessed 922, 2020

[5] National Association of Community Health Center. PRAPARE—NACHC. 2020. Available at http://www.nachc.org/research-and-data/prapare/ Accessed 61, 2020

[6] Centers for Medicare and Medicaid Services, Billioux A, Verlander K, et al. Standardized screening for health-related social needs in clinical settings: the accountable health communities screening tool. NAM Perspectives. 2017;7

[7] Beck AF, Henize AW, Kahn RS, et al. Forging a pediatric primary care–community partnership to support food-insecure families. Pediatrics. 2014;134:e564–e571 25049345 10.1542/peds.2013-3845

[8] Weintraub D, Rodgers MA, Botcheva L, et al. Pilot study of medical-legal partnership to address social and legal needs of patients. J Health Care Poor Underserved. 2010;21(2 Suppl):157–168 20453383 10.1353/hpu.0.0311

[9] The National Academies of Sciences, Engineering, and Medicine. Integrating Social Care into the Delivery of Health Care: Moving Upstream to Improve the Nation's Health. Washington, DC: The National Academies Press, 2019 31940159

[10] AAP Committee on Fetus and Newborn and AC OG Committee on Obstetric Practice. Guidelines for Perinatal Care, 8th Edition. Washington, DC: American College of Obstetricians and Gynecologists, 2017

[11] American College of Obstetricians and Gynecologists. Racial and Ethnic Disparities in Obstetrics and Gynecology. 2020. Available at https://www.acog.org/en/Clinical/Clinical Guidance/Committee Accessed 61, 2020

[12] Nussey L, Hunter A, Krueger S, et al. Sociodemographic characteristics and clinical outcomes of people receiving inadequate prenatal care: a retrospective cohort study. J Obstet Gynaecol Can. 2020;42:591–600 31818693 10.1016/j.jogc.2019.08.005

[13] Osterman MJK. Timing and Adequacy of Prenatal Care in the United States, 2016. National Vital Statistics Reports; vol. 67. Hyattsville, MD: National Center for Health Statistics, 2020 29874159

[14] Heaman MI, Sword W, Elliott L, et al. Barriers and facilitators related to use of prenatal care by inner-city women: perceptions of health care providers. BMC Pregnancy Childbirth. 2015;15:2 25591945 10.1186/s12884-015-0431-5 PMC4302607

[15] Mazul MC, Salm Ward TC, Ngui EM. Anatomy of good prenatal care: perspectives of low income African-American women on barriers and facilitators to prenatal care. J Racial Ethn Health Disparities. 2017;4:79–86 26823064 10.1007/s40615-015-0204-x

[16] Tucker Edmonds B, Mogul M, Shea JA. Understanding low-income African American women's expectations, preferences, and priorities in prenatal care. Fam Community Health. 2015;38:149–157 25739062 10.1097/FCH.0000000000000066

[17] Gottlieb LM, Wing H, Adler NE. A systematic review of interventions on patients' social and economic needs. Am J Prev Med. 2017;53:719–729 28688725 10.1016/j.amepre.2017.05.011

[18] Andermann A. Screening for social determinants of health in clinical care: moving from the margins to the mainstream. Public Health Rev. 2018;39:19 29977645 10.1186/s40985-018-0094-7 PMC6014006

[19] Ouzzani M, Hammady H, Fedorowicz Z, et al. Rayyan-a web and mobile app for systematic reviews. Syst Rev. 2016;5:210 27919275 10.1186/s13643-016-0384-4 PMC5139140

[20] US Department of Health and Human Services. Social Determinants of Health | Healthy People 2020. 2014. Available at https://www.healthypeople.gov/2020/topics-objectives/topic/social-determinants-of-health Accessed 61, 2020

[21] Krist AH, Davidson KW, Ngo-Metzger Q. What evidence do we need before recommending routine screening for social determinants of health? Am Fam Physician 2019;99:602–605 31083876

[22] The World Bank. World Bank Country and Lending Groups. Available at https://datahelpdesk.worldbank.org/knowledgebase/articles/906519-world-bank-country-and-lending-groups Accessed 61, 2020

[23] Guyatt GH, Oxman AD, Vist GE, et al. GRADE: an emerging consensus on rating quality of evidence and strength of recommendations. BMJ. 2008;336:924–926 18436948 10.1136/bmj.39489.470347.AD PMC2335261

[24] Guyatt GH, Oxman AD, Vist G, et al. GRADE guidelines: 4. Rating the quality of evidence—study limitations (risk of bias). J Clin Epidemiol. 2011;64:407–415 21247734 10.1016/j.jclinepi.2010.07.017

[25] El-Mohandes AAE, Kiely M, Joseph JG, et al. An integrated intervention in pregnant African Americans reduces postpartum risk: a randomized trial. Obstet Gynecol. 2008;112:611–620 18757660 10.1097/AOG.0b013e3181834b10 PMC2935657

[26] El-Mohandes AAE, Kiely M, Blake SM, et al. An intervention to reduce environmental tobacco smoke exposure improves pregnancy outcomes. Pediatrics. 2010;125:721–728 20211945 10.1542/peds.2009-1809 PMC2923806

[27] El-Mohandes AAE, Kiely M, Gantz MG, et al. Very preterm birth is reduced in women receiving an integrated behavioral intervention: a randomized controlled trial. Matern Child Health J. 2011;15:19–28 20082130 10.1007/s10995-009-0557-z PMC2988881

[28] Kiely M, Gantz M, El-Khorazaty M, et al. Sequential screening for psychosocial and behavioural risk during pregnancy in a population of urban African Americans. BJOG. 2013;120:1395–1402 23906260 10.1111/1471-0528.12202 PMC3775859

[29] Kiely M, El-Mohandes AAE, El-Khorazaty MN, et al. An integrated intervention to reduce intimate partner violence in pregnancy: a randomized controlled trial. Obstet Gynecol. 2010;115(2, Part 1):273–283 20093899 10.1097/AOG.0b013e3181cbd482 PMC2917915

[30] Katz KS, Blake SM, Milligan RA, et al. The design, implementation and acceptability of an integrated intervention to address multiple behavioral and psychosocial risk factors among pregnant African American women. BMC Pregnancy Childbirth. 2008;8:22 18578875 10.1186/1471-2393-8-22 PMC2474573

[31] Backonja U, Robledo CA, Wallace ME, et al. Reproductive health knowledge among African American wome3n enrolled in a clinic-based randomized controlled trial to reduce psychosocial and behavioral risk: project DC-HOPE. Womens Health Issues. 2016;26:442–451 27094910 10.1016/j.whi.2016.03.005 PMC4958531

[32] Calderón SH, Gilbert P, Jackson R, et al. Cueing prenatal providers. Am J Prev Med. 2008;34:134–137 18201643 10.1016/j.amepre.2007.09.029 PMC2242423

[33] Curry MA, Durham L, Bullock L, et al. Nurse case management for pregnant women experiencing or at risk for abuse. J Obstet Gynecol Neonatal Nurs. 2006;35:181–192 10.1111/j.1552-6909.2006.00027.x 16620243

[34] Humphreys J, Tsoh JY, Kohn MA, et al. Increasing discussions of intimate partner violence in prenatal care using video doctor plus provider cueing: a randomized, controlled trial. Womens Health Issues. 2011;21:136–144 21185737 10.1016/j.whi.2010.09.006 PMC3053017

[35] McFarlane J, Soeken K, Wiist W. An evaluation of interventions to decrease intimate partner violence to pregnant women. Public Health Nursing. 2000;17:443–451 11115142 10.1046/j.1525-1446.2000.00443.x

[36] Sayakhot P, Carolan-Olah M, Steele C. Use of a web-based educational intervention to improve knowledge of healthy diet and lifestyle in women with gestational diabetes mellitus compared to standard clinic-based education. BMC Pregnancy Childbirth. 2016;16:208 27495978 10.1186/s12884-016-0996-7 PMC4974775

[37] Tiwari A, Leung WC, Leung TW, et al. A randomised controlled trial of empowerment training for Chinese abused pregnant women in Hong Kong: empowerment training for Chinese abused pregnant women. BJOG. 2005;112:1249–1256 16101604 10.1111/j.1471-0528.2005.00709.x

[38] Van Parys A-S, Deschepper E, Roelens K, et al. The impact of a referral card-based intervention on intimate partner violence, psychosocial health, help-seeking and safety behaviour during pregnancy and postpartum: a randomized controlled trial. BMC Pregnancy Childbirth. 2017;17:346 28985722 10.1186/s12884-017-1519-x PMC6389099

[39] Zlotnick C, Capezza NM, Parker D. An interpersonally based intervention for low-income pregnant women with intimate partner violence: a pilot study. Arch Womens Ment Health. 2011;14:55–65 21153559 10.1007/s00737-010-0195-x PMC3042850

[40] Bernazzani O, Marks MN, Bifulco A, et al. Assessing psychosocial risk in pregnant/postpartum women using the Contextual Assessment of Maternity Experience (CAME)—recent life adversity, social support and maternal feelings. Soc Psychiatry Psychiatr Epidemiol. 2005;40:497–508 16003600 10.1007/s00127-005-0917-y

[41] Chang JC, Dado D, Schussler S, et al. In person versus computer screening for intimate partner violence among pregnant patients. Patient Educ Couns. 2012;88:443–448 22770815 10.1016/j.pec.2012.06.021 PMC3413751

[42] Connelly CD, Newton RR, Landsverk J, et al. Assessment of intimate partner violence among high-risk postpartum mothers: concordance of clinical measures. Women Health. 2000;31:21–37 10.1300/J013v31n01_02 11005218

[43] Kramer A, Nosbusch JM, Rice J. Safe Mom, Safe Baby: a collaborative model of care for pregnant women experiencing intimate partner violence. J Perinat Neonatal Nurs. 2012;26:307–316 23111718 10.1097/JPN.0b013e31824356dd

[44] Priest SR, Austin M-P, Barnett BB, et al. A psychosocial risk assessment model (PRAM) for use with pregnant and postpartum women in primary care settings. Arch Womens Ment Health. 2008;11:307–317 18726142 10.1007/s00737-008-0028-3

[45] Trapl ES, Joshi K, Taggart M, et al. Mixed methods evaluation of a produce prescription program for pregnant women. J Hunger Environ Nutr. 2017;12:529–543

[46] Price SK, Coles DC, Wingold T. Integrating behavioral health risk assessment into centralized intake for maternal and child health services. Health Soc Work. 2017;42:231–240 29025051 10.1093/hsw/hlx037

[47] Carroll JC. Effectiveness of the Antenatal Psychosocial Health Assessment (ALPHA) form in detecting psychosocial concerns: a randomized controlled trial. Can Med Assoc J. 2005;173:253–259 16076821 10.1503/cmaj.1040610 PMC1180654

[48] Gance-Cleveland B, Leiferman J, Aldrich H, et al. Using the technology acceptance model to develop *StartSmart*: mHealth for screening, brief Intervention, and referral for risk and protective factors in pregnancy. J Midwifery Womens Health. 2019;64:630–640 31347784 10.1111/jmwh.13009

[49] Zachek CM, Schwartz JM, Glasser M, et al. A screening questionnaire for occupational and hobby exposures during pregnancy. Occup Med. 2019;69:428–435 10.1093/occmed/kqz094 PMC6939873 31247109

[50] Price A, Bryson H, Mensah F, et al. A brief survey to identify pregnant women experiencing increased psychosocial and socioeconomic risk. Women Birth. 2019;32:e351–e358 30193913 10.1016/j.wombi.2018.08.162

[51] Harrison PA, Sidebottom AC. Systematic prenatal screening for psychosocial risks. J Health Care Poor Underserved. 2008;19:258–276 18264001 10.1353/hpu.2008.0003

[52] Watt TT, Appel L, Lopez V, et al. A primary care-based early childhood nutrition intervention: evaluation of a pilot program serving low-income hispanic women. J Racial Ethn Health Disparities. 2015;2:537–547 26863560 10.1007/s40615-015-0102-2

[53] Cohen D, Lisles C, Williams WR, et al. Exploratory study to evaluate the provision of additional midwifery support to teenage mothers. Public Health. 2011;125:632–638 21855098 10.1016/j.puhe.2011.06.008

[54] Taft AJ, Hooker L, Humphreys C, et al. Maternal and child health nurse screening and care for mothers experiencing domestic violence (MOVE): a cluster randomised trial. BMC Med. 2015;13:150 26111528 10.1186/s12916-015-0375-7 PMC4480893

[55] Parker B, McFarlane J, Soeken K, et al. Testing an intervention to prevent further abuse to pregnant women. Res Nurs Health. 1999;22:59–66 9928964 10.1002/(sici)1098-240x(199902)22:1<59::aid-nur7>3.0.co;2-b

[56] US Preventive Services Task Force. Report of the Task Force on Screening for Intimate Partner Violence, Elder Abuse, and Abuse of Vulnerable Adults. 2018. Rockville, MD, US Preventive Services Task Force

[57] Driscoll AK, Osterman MJK. Maternal characteristics of prenatal WIC receipt in the United States, 2016. NCHS Data Brief. 2018:1–8 29442995

[58] Sonchak L. The impact of WIC on birth outcomes: new evidence from South Carolina. Matern Child Health J. 2016;20:1518–1525 26976280 10.1007/s10995-016-1951-y

[59] Bitler MP, Currie J. Does WIC work? The effects of WIC on pregnancy and birth outcomes. J Policy Anal Manage. 2005;24:73–91 15584177 10.1002/pam.20070

[60] Hamad R, Collin DF, Baer RJ, et al. Association of revised WIC food package with perinatal and birth outcomes: a quasi-experimental study. JAMA Pediatr. 2019;173:845–852 10.1001/jamapediatrics.2019.1706 PMC6604113 31260072

[61] Kowaleski-Jones L, Duncan GJ. Effects of participation in the WIC program on birthweight: evidence from the National Longitudinal Survey of Youth. Am J Public Health. 2002;92:799–804 11988450 10.2105/ajph.92.5.799 PMC1447164

[62] Soneji S, Beltran-Sanchez H. Association of special supplemental nutrition program for women, infants, and children with preterm birth and infant mortality. JAMA Netw Open. 2019;2:e1916722 31800070 10.1001/jamanetworkopen.2019.16722 PMC6902759

[63] Gibson-Helm M, Boyle J, Cheng I-H, et al. Maternal health and pregnancy outcomes among women of refugee background from Asian countries. Int J Gynecol Obstet. 2015;129:146–151 10.1016/j.ijgo.2014.10.036 25640714

[64] Carolan M. Pregnancy health status of sub-Saharan refugee women who have resettled in developed countries: a review of the literature. Midwifery. 2010;26:407–414 19121552 10.1016/j.midw.2008.11.002

[65] Leonard SA, Main EK, Scott KA, et al. Racial and ethnic disparities in severe maternal morbidity prevalence and trends. Ann Epidemiol. 2019;33:30–36 30928320 10.1016/j.annepidem.2019.02.007 PMC6502679

[66] The National Academies of Sciences, Engineering, and Medicine. Birth Settings in America: Outcomes, Quality, Access, and Choice. Washington, DC: The National Academies Press, 2020 32049472

