# Supplementary material for: Interventions Addressing Social Needs in Perinatal Care: A Systematic Review
Source: Health Equity. 2021 Mar 4;5(1):100–18. doi: 10.1089/heq.2020.0051 (PMC7990569; doi:10.1089/heq.2020.0051)
Supplement: Supplemental data [file Supp_AppendixS1.docx]

**Reyes Search Strategies SDH Pregnancy Systematic Review**

**PubMed:**

6/26/2019, repeated 5/6/2020

((((“health planning"[Mesh] OR "health communication"[Mesh] OR “health services research”[Mesh] OR “delivery of health care”[Mesh] OR “delivery of care”[TIAB] OR “financial”[TIAB] OR “housing”[TIAB] OR “food insecurity”[TIAB] OR “childcare”[TIAB] OR “child care”[TIAB] OR “intimate partner violence”[TIAB] OR “safety”[TIAB] OR “social work”[TIAB] OR “social worker”[TIAB] OR “WIC”[TIAB] OR “Supplemental Nutrition Program”[TIAB] OR “family case management”[TIAB] OR “education status”[TIAB] OR “social welfare”[TIAB] OR employ*[TIAB] OR unemploy*[TIAB] OR “food assistance”[TIAB] OR “food supply”[TIAB] OR “hunger”[TIAB] OR “domestic violence”[TIAB] OR “social isolation”[TIAB] OR “transportation”[TIAB] OR “Psychosocial Deprivation”[Mesh] OR "Psychosocial risk"[TIAB]))) AND ((Barrier*[TIAB] OR Bias*[TIAB] OR “delivery of health care”[Mesh] OR “delivery of care”[TIAB] OR dispar*[TIAB] OR disproportionate*[TIAB] OR “health behavior”[Mesh] OR “health knowledge, attitudes, practice”[Mesh] OR “health services accessibility”[Mesh] OR “health services, indigenous”[Mesh] OR “health services needs and demand”[Mesh] OR inequali*[TIAB] OR inequit*[TIAB] OR “patient acceptance of health care”[Mesh] OR “patient selection”[Mesh] OR “quality of health care”[MeSH] OR “culturally competent care”[Mesh] OR “culturally competent care”[TIAB] OR “socioeconomic factor”[TIAB] OR “socioeconomic factors”[Mesh] OR “socioeconomic factors”[TIAB] OR “socioeconomically disadvantaged”[TIAB] OR “Social Determinants of Health”[Mesh] OR underrepresent*[TIAB] OR underserve*[TIAB]) OR “ethnic disparities”[TIAB] OR “ethnic disparity”[TIAB] OR “health disparities”[TIAB] OR “health disparity”[TIAB] OR “healthcare disparities”[Mesh] OR “health care disparities”[TIAB] OR “healthcare disparities”[TIAB] OR “health-care disparities”[TIAB] OR “health care disparity”[TIAB] OR “healthcare disparity”[TIAB] OR “health-care disparity”[TIAB] OR “health status disparities”[Mesh] OR “disparities in care”[TIAB]) OR (“J Health Care Poor Underserved”[Journal] OR “J Health Dispar Res Pract”[Journal] OR “J Racial Ethn Health Disparities”[Journal]) OR “culturally competent care”[Mesh] OR “health behavior”[TIAB] OR “health behaviors”[TIAB] OR “health inequality”[TIAB] OR “health inequalities”[TIAB] OR “health inequities”[TIAB] OR “health inequity”[TIAB] OR “health related quality of life”[TIAB] OR “health-related quality of life”[TIAB] OR “health status disparities”[Mesh] OR “social class”[Mesh] OR “social class”[TIAB] OR “social determinants of health”[TIAB] OR “social disparities”[TIAB] OR “social disparity”[TIAB] OR “social factors”[TIAB] OR “social inequities”[TIAB] OR “social inequity”[TIAB] OR at-risk [TIAB] OR “at risk”[TIAB] OR high-risk [TIAB] OR “high risk”[TIAB]) AND ((“Health Surveys”[Mesh] OR “Screening tools”[TIAB] OR “screening tool”[TIAB] OR “Assessment” [TIAB] OR “assessments”[TIAB] OR “Referral and Consultation”[Mesh] OR “patient education”[TIAB] OR “patient educator”[TIAB] OR “patient educators”[TIAB] OR “mass screening”[TIAB] OR “mass screenings”[TIAB] OR “Crisis Intervention”[Mesh] OR "early medical intervention"[MeSH] OR ("early"[TIAB] AND "medical"[TIAB] AND "intervention"[TIAB]) OR "early medical intervention"[TIAB] OR “Risk Assessment”[Mesh] OR “risk assessment”[TIAB])) AND (((“Maternal Care” [TIAB] OR “Maternal Health”[Mesh] OR “Maternal Health Services”[Mesh] OR “Perinatal”[TIAB] OR “Prenatal”[TIAB] OR “Antenatal”[TIAB] OR “Postpartum”[TIAB] OR “Pregnancy”[Mesh] OR “Pregnant Women”[Mesh] OR Pregnant*[TIAB] OR Pregnanc*[TIAB] OR obstetric*[TIAB]))) AND ((((“Health outcomes”[TIAB] OR (("health"[MeSH Terms] OR health[TIAB]) AND outcomes[TIAB]) OR “Pregnancy Complications”[Mesh] OR “pregnancy complications”[TIAB] OR ((“pregnancy”[Mesh] OR pregnancy [TIAB]) AND (complication[TIAB] OR complications[TIAB])) OR “Fetal death” [TIAB] OR stillbirth[TIAB] OR stillborn[TIAB] OR “abortion, spontaneous”[Mesh] OR “spontaneous abortion” OR “pregnancy loss”[TIAB] OR “pregnancy losses”[TIAB] OR Miscarriage*[TIAB] OR Miscarry[TIAB] OR ((medical[TIAB] OR health[TIAB]) AND (Complication[TIAB] OR complications[TIAB])) OR "Maternal Mortality"[Mesh] OR (maternal[TIAB] AND mortalit*[TIAB]) OR “infant, low birth weight” [Mesh] OR “low birth weight” [TIAB] OR LBW[TIAB] OR “preterm birth” [TIAB] OR “premature birth”[TIAB] OR “premature labor”[TIAB] OR “preterm labor”[TIAB] OR “premature labour”[TIAB] OR “preterm labour”[TIAB])))) AND (((((((((“health planning"[Mesh] OR "health communication"[Mesh] OR “health services research”[Mesh] OR “delivery of health care”[Mesh] OR “delivery of care”[TIAB] OR “financial”[TIAB] OR “housing”[TIAB] OR “food insecurity”[TIAB] OR “childcare”[TIAB] OR “child care”[TIAB] OR “intimate partner violence”[TIAB] OR “safety”[TIAB] OR “social work”[TIAB] OR “social worker”[TIAB] OR “WIC”[TIAB] OR “Supplemental Nutrition Program”[TIAB] OR “family case management”[TIAB] OR “education status”[TIAB] OR “social welfare”[TIAB] OR employ*[TIAB] OR unemploy*[TIAB] OR “food assistance”[TIAB] OR “food supply”[TIAB] OR “hunger”[TIAB] OR “domestic violence”[TIAB] OR “social isolation”[TIAB] OR “transportation”[TIAB] OR “Psychosocial Deprivation”[Mesh] OR "Psychosocial risk"[TIAB]))))) AND ((((Barrier*[TIAB] OR Bias*[TIAB] OR “delivery of health care”[Mesh] OR “delivery of care”[TIAB] OR dispar*[TIAB] OR disproportionate*[TIAB] OR “health behavior”[Mesh] OR “health knowledge, attitudes, practice”[Mesh] OR “health services accessibility”[Mesh] OR “health services, indigenous”[Mesh] OR “health services needs and demand”[Mesh] OR inequali*[TIAB] OR inequit*[TIAB] OR “patient acceptance of health care”[Mesh] OR “patient selection”[Mesh] OR “quality of health care”[MeSH] OR “culturally competent care”[Mesh] OR “culturally competent care”[TIAB] OR “socioeconomic factor”[TIAB] OR “socioeconomic factors”[Mesh] OR “socioeconomic factors”[TIAB] OR “socioeconomically disadvantaged”[TIAB] OR “Social Determinants of Health”[Mesh] OR underrepresent*[TIAB] OR underserve*[TIAB]) OR “ethnic disparities”[TIAB] OR “ethnic disparity”[TIAB] OR “health disparities”[TIAB] OR “health disparity”[TIAB] OR “healthcare disparities”[Mesh] OR “health care disparities”[TIAB] OR “healthcare disparities”[TIAB] OR “health-care disparities”[TIAB] OR “health care disparity”[TIAB] OR “healthcare disparity”[TIAB] OR “health-care disparity”[TIAB] OR “health status disparities”[Mesh] OR “disparities in care”[TIAB]) OR (“J Health Care Poor Underserved”[Journal] OR “J Health Dispar Res Pract”[Journal] OR “J Racial Ethn Health Disparities”[Journal]) OR “culturally competent care”[Mesh] OR “health behavior”[TIAB] OR “health behaviors”[TIAB] OR “health inequality”[TIAB] OR “health inequalities”[TIAB] OR “health inequities”[TIAB] OR “health inequity”[TIAB] OR “health related quality of life”[TIAB] OR “health-related quality of life”[TIAB] OR “health status disparities”[Mesh] OR “social class”[Mesh] OR “social class”[TIAB] OR “social determinants of health”[TIAB] OR “social disparities”[TIAB] OR “social disparity”[TIAB] OR “social factors”[TIAB] OR “social inequities”[TIAB] OR “social inequity”[TIAB] OR at-risk [TIAB] OR “at risk”[TIAB] OR high-risk [TIAB] OR “high risk”[TIAB])))) AND ((((“Health Surveys”[Mesh] OR “Screening tools”[TIAB] OR “screening tool”[TIAB] OR “Assessment” [TIAB] OR “assessments”[TIAB] OR “Referral and Consultation”[Mesh] OR “patient education”[TIAB] OR “patient educator”[TIAB] OR “patient educators”[TIAB] OR “mass screening”[TIAB] OR “mass screenings”[TIAB] OR “Crisis Intervention”[Mesh] OR "early medical intervention"[MeSH] OR ("early"[TIAB] AND "medical"[TIAB] AND "intervention"[TIAB]) OR "early medical intervention"[TIAB] OR “Risk Assessment”[Mesh] OR “risk assessment”[TIAB]))))) AND (((((“Maternal Care” [TIAB] OR “Maternal Health”[Mesh] OR “Maternal Health Services”[Mesh] OR “Perinatal”[TIAB] OR “Prenatal”[TIAB] OR “Antenatal”[TIAB] OR “Postpartum”[TIAB] OR “Pregnancy”[Mesh] OR “Pregnant Women”[Mesh] OR Pregnant*[TIAB] OR Pregnanc*[TIAB] OR obstetric*[TIAB])))))) AND ((((((“Health outcomes”[TIAB] OR (("health"[MeSH Terms] OR health[TIAB]) AND outcomes[TIAB]) OR “Pregnancy Complications”[Mesh] OR “pregnancy complications”[TIAB] OR ((“pregnancy”[Mesh] OR pregnancy [TIAB]) AND (complication[TIAB] OR complications[TIAB])) OR “Fetal death” [TIAB] OR stillbirth[TIAB] OR stillborn[TIAB] OR “abortion, spontaneous”[Mesh] OR “spontaneous abortion” OR “pregnancy loss”[TIAB] OR “pregnancy losses”[TIAB] OR Miscarriage*[TIAB] OR Miscarry[TIAB] OR ((medical[TIAB] OR health[TIAB]) AND (Complication[TIAB] OR complications[TIAB])) OR "Maternal Mortality"[Mesh] OR (maternal[TIAB] AND mortalit*[TIAB]) OR “infant, low birth weight” [Mesh] OR “low birth weight” [TIAB] OR LBW[TIAB] OR “preterm birth” [TIAB] OR “premature birth”[TIAB] OR “premature labor”[TIAB] OR “preterm labor”[TIAB] OR “premature labour”[TIAB] OR “preterm labour”[TIAB])))))) 
**N=6221, N=678**

**Embase** 
6/26/2019, repeated 5/6/2020

#1 - 'health care planning'/exp OR 'health services research'/exp OR 'health care delivery'/exp OR 'social isolation'/exp OR ‘health planning’ OR  ‘health communication’ OR ‘health services research’ OR ‘delivery of health care’ OR ‘delivery of care’ OR ‘financial’ OR ‘housing’ OR ‘food insecurity’ OR ‘childcare’ OR ‘child care’ OR ‘intimate partner violence’ OR ‘safety’ OR ‘social work’ OR ‘social workers’ OR ‘social worker’ OR ‘WIC’ OR ‘supplemental nutrition program’ OR ‘family case management’ OR ‘education status’ OR ‘social welfare’ OR employ* OR unemploy* OR 'food assistance’ OR 'food supply’ OR ‘hunger’ OR ‘domestic violence’ OR ‘social isolation’ OR ‘transportation’ OR ‘Psychosocial Deprivation’ OR ‘Psychosocial risk’ 

#2 - 'health care delivery'/exp OR 'health behavior'/exp OR 'attitude to health'/exp OR 'indigenous health care'/exp OR 'patient attitude'/exp OR 'patient selection'/exp OR 'health care quality'/exp OR 'transcultural care'/exp OR 'socioeconomics'/exp OR 'social determinants of health'/exp OR 'health care disparity'/exp OR 'health disparity'/exp OR 'social class'/exp OR disproportionate* OR ‘health behavior’ OR ‘health knowledge attitudes practices’ OR ‘health services accessibility’ OR ‘health services indigenous’ OR ‘indigenous health services’ OR ‘health services needs and demands’ OR inequali* OR inequit* OR ‘patient acceptance of health care’ OR ‘patient selection’ OR ‘quality of health care’ OR ‘culturally competent care’ OR ‘socioeconomic factors’ OR ‘socioeconomically disadvantaged’ OR ‘social determinants of health’ OR underrepresent* OR underserve* OR ‘ethnic disparities’ OR ‘ethnic disparity’ OR ‘health disparities’ OR ‘health disparity’ OR ‘healthcare disparities’ OR ‘healthcare disparities’ OR ‘health-care disparities’ OR ‘health care disparity’ OR ‘healthcare disparity’ OR ‘health-care disparity’ OR ‘health status disparities’ OR ‘disparities in care’ OR ‘health behavior’ OR ‘health behaviors’ OR ‘health inequality’ OR ‘health inequalities’ OR ‘health inequities’ OR ‘health inequity’ OR ‘health related quality of life’ OR ‘health-related quality of life’ OR ‘health status disparities’ OR ‘social class’ OR ‘social disparities’ OR ‘social disparity’ OR ‘social factors’ OR ‘social inequities’ OR ‘social inequality’ OR ‘social inequalities’ OR ‘social inequity’  OR ‘at risk’  OR ‘high risk’ 

#3 - 'health survey'/exp OR 'patient referral'/exp OR 'crisis intervention'/exp OR 'early intervention'/exp OR 'risk assessment'/exp OR 'health surveys' OR 'screening tools' OR 'screening tool' OR 'assessment' OR 'assessments' OR 'referral' OR 'referred' OR 'refers' OR 'refer' OR 'patient education' OR 'patient educator' OR 'patient educators' OR 'mass screening' OR 'mass screenings' OR 'crisis intervention' OR 'early medical intervention' OR 'risk assessment'  

#4 - 'maternal welfare'/exp OR 'maternal health service'/exp OR 'pregnancy'/exp OR 'maternal care'/exp OR 'pregnant woman'/exp OR 'maternal care' OR 'maternal health' OR perinatal OR prenatal OR antenatal OR postpartum OR pregnancy OR 'pregnant women' OR pregnant* OR pregnanc* OR obstetric* 

#5 - 'pregnancy complication'/exp OR 'spontaneous abortion'/exp OR 'maternal mortality'/exp OR 'low birth weight'/exp OR 'health outcomes' OR 'pregnancy complications' OR ‘pregnancy complication’ OR 'fetal death' OR stillbirth OR stillborn OR 'spontaneous abortion' OR 'pregnancy loss' OR 'pregnancy losses' OR miscarriage* OR miscarry OR ‘medical complication*’ OR ‘health complication*’ OR 'maternal mortalit*' OR 'low birth weight' OR lbw OR 'preterm birth' OR 'premature birth' OR 'premature labor' OR 'preterm labor' OR 'premature labour' OR 'preterm labour'

#1 AND #2 AND #3 AND #4 AND #5

**N=13,996**, **N=1,598**

**Cochrane** 
6/26/2019, repeated 5/6/2020

#1 – MeSH descriptor: [health planning] explode all trees

#2 – MeSH descriptor: [health communication] explode all trees

#3 - MeSH descriptor: [health services research] explode all trees

#4 - MeSH descriptor: [delivery of health care] explode all trees

#5 - MeSH descriptor: [Psychosocial Deprivation] explode all trees

#6 - ("health planning" OR "health communication" OR "health services research" OR "delivery of health care" OR "delivery of care" OR "financial" OR "housing" OR "food insecurity" OR "childcare" OR "child care" OR "intimate partner violence" OR "safety" OR "social work" OR "social workers" OR "social worker" OR "WIC" OR "supplemental nutrition program" OR "family case management" OR "education status" OR "social welfare" OR employ* OR unemploy* OR "food assistance" OR "food supply" OR "hunger" OR "domestic violence" OR "social isolation" OR "transportation" OR "Psychosocial Deprivation" OR "Psychosocial risk"):ti,ab,kw

**#7 -** #1 OR #2 OR #3 OR #4 OR #5 OR #6

#8 - MeSH descriptor: [delivery of health care] explode all trees

#9 - MeSH descriptor: [health behavior] explode all trees

#10 - MeSH descriptor: [health knowledge, attitudes, practice] explode all trees

#11 - MeSH descriptor: [health services accessibility] explode all trees

#12 - MeSH descriptor: [health services, indigenous] explode all trees

#13 - MeSH descriptor: [health services needs and demand] explode all trees

#14 - MeSH descriptor: [patient acceptance of health care] explode all trees

#15 - MeSH descriptor: [patient selection] explode all trees

#16 - MeSH descriptor: [quality of health care] explode all trees

#17 - MeSH descriptor: [culturally competent care] explode all trees

#18 - MeSH descriptor: [socioeconomic factors] explode all trees

#19 - MeSH descriptor: [Social Determinants of Health] explode all trees

#20 - MeSH descriptor: [healthcare disparities] explode all trees

#21 - MeSH descriptor: [health status disparities] explode all trees

#22 - MeSH descriptor: [social class] explode all trees

#23 - (barrier* OR bias* OR “delivery of health care” OR “delivery of care” OR dispar* OR disproportionate* OR “health behavior” OR “health knowledge attitudes practices” OR “health services accessibility” OR “health services indigenous” OR “indigenous health services” OR “health services needs and demands” OR inequali* OR inequit* OR “patient acceptance of health care” OR “patient selection” OR “quality of health care” OR “culturally competent care” OR “socioeconomic factors” OR “socioeconomically disadvantaged” OR “social determinants of health” OR underrepresent* OR underserve* OR “ethnic disparities” OR “ethnic disparity” OR “health disparities” OR “health disparity” OR “healthcare disparities” OR “healthcare disparities” OR “health-care disparities” OR “health care disparity” OR “healthcare disparity” OR “health-care disparity” OR “health status disparities” OR “disparities in care” OR “health behavior” OR “health behaviors” OR “health inequality” OR “health inequalities” OR “health inequities” OR “health inequity” OR “health related quality of life” OR “health-related quality of life” OR “health status disparities” OR “social class” OR “social disparities” OR “social disparity” OR “social factors” OR “social inequities” OR “social inequality” OR “social inequalities” OR “social inequity” OR at-risk OR “at risk” OR high-risk OR “high risk”):ti,ab,kw

**#24** - #8 OR … #23

#25 - MeSH descriptor: [Health Surveys] explode all trees

#26 - MeSH descriptor: [Referral and Consultation] explode all trees

#27 - MeSH descriptor: [Crisis Intervention] explode all trees

#28 - MeSH descriptor: [early medical intervention] explode all trees

#29 - MeSH descriptor: [Risk Assessment] explode all trees

#30 - ((“Health Surveys” OR “Screening tools” OR “screening tool” OR “Assessment” OR “assessments” OR “Referral and Consultation” OR “patient education” OR “patient educator” OR “patient educators” OR “mass screening” OR “mass screenings” OR “Crisis Intervention” OR "early medical intervention" OR ("early" AND "medical" AND "intervention") OR “risk assessment”)):ti,ab,kw 
**#31** - #25 OR …#30

#32- MeSH descriptor: [Maternal Health] explode all trees

#33 - MeSH descriptor: [Maternal Health Services] explode all trees

#34 - MeSH descriptor: [Pregnancy] explode all trees

#35 - MeSH descriptor: [Pregnant Women] explode all trees 
#36 - (“Maternal Care” OR “Maternal Health” OR Perinatal OR Prenatal OR Antenatal OR Postpartum OR Pregnancy OR “Pregnant Women” OR Pregnant* OR Pregnanc* OR obstetric*):ti,ab,kw

**#37** - #32 OR … #36

#38 - MeSH descriptor: [Pregnancy Complications] explode all trees

#39 - MeSH descriptor: [abortion, spontaneous] explode all trees

#40 - MeSH descriptor: [Maternal Mortality] explode all trees

#41 - MeSH descriptor: [infant, low birth weight] explode all trees 
#42 - (“Health outcomes” OR (health AND outcomes) OR “Pregnancy Complications” OR (pregnancy AND (complication OR complications)) OR “Fetal death” OR stillbirth OR stillborn OR “spontaneous abortion” OR “pregnancy loss” OR “pregnancy losses” OR Miscarriage* OR Miscarry OR ((medical OR health) AND (Complication OR complications)) OR "Maternal Mortality" OR (maternal AND mortalit*) OR “low birth weight” OR LBW OR “preterm birth” OR “premature birth” OR “premature labor” OR “preterm labor” OR “premature labour” OR “preterm labour”):ti,ab,kw 
**#43** - #38 OR … #42

#44 - #7 AND #24 AND #31 AND #37 AND #43

**N=1243, N=190**

**CINAHL** 
6/27/2019, repeated 5/6/2020

S1 – (MH "Health and Welfare Planning+") OR (MH "Health Services Research+") OR (MH "Health Care Delivery+") OR (MH "Psychosocial Deprivation")

S2 – ("health planning" OR "health communication" OR "health services research" OR "delivery of health care" OR "delivery of care" OR "financial" OR "housing" OR "food insecurity" OR "childcare" OR "child care" OR "intimate partner violence" OR "safety" OR "social work" OR "social workers" OR "social worker" OR "WIC" OR "supplemental nutrition program" OR "family case management" OR "education status" OR "social welfare" OR employ* OR unemploy* OR "food assistance" OR "food supply" OR "hunger" OR "domestic violence" OR "social isolation" OR "transportation" OR "Psychosocial Deprivation" OR "Psychosocial risk")

**S3 – S1 OR S2**

S4 - (MH "Health Care Delivery+") OR (MH "Health Behavior+") OR (MH "Health Knowledge") OR (MH "Health Services Accessibility+") OR (MH "Health Services Needs and Demand+") OR (MH "Health Services, Indigenous") OR (MH "Patient Selection") OR (MH "Quality of Health Care+") OR (MH "Cultural Competence") OR (MH "Transcultural Care") OR (MH "Socioeconomic Factors+") OR (MH "Health Status+") OR (MH "Social Determinants of Health") OR (MH "Healthcare Disparities") OR (MH "Health Status Disparities") OR (MH "Social Class+")

S5 - (barrier* OR bias* OR “delivery of health care” OR “delivery of care” OR dispar* OR disproportionate* OR “health behavior” OR “health knowledge attitudes practices” OR “health services accessibility” OR “health services indigenous” OR “indigenous health services” OR “health services needs and demands” OR inequali* OR inequit* OR “patient acceptance of health care” OR “patient selection” OR “quality of health care” OR “culturally competent care” OR “socioeconomic factors” OR “socioeconomically disadvantaged” OR “social determinants of health” OR underrepresent* OR underserve* OR “ethnic disparities” OR “ethnic disparity” OR “health disparities” OR “health disparity” OR “healthcare disparities” OR “healthcare disparities” OR “health-care disparities” OR “health care disparity” OR “healthcare disparity” OR “health-care disparity” OR “health status disparities” OR “disparities in care” OR “health behavior” OR “health behaviors” OR “health inequality” OR “health inequalities” OR “health inequities” OR “health inequity” OR “health related quality of life” OR “health-related quality of life” OR “health status disparities” OR “social class” OR “social disparities” OR “social disparity” OR “social factors” OR “social inequities” OR “social inequality” OR “social inequalities” OR “social inequity” OR at-risk OR “at risk” OR high-risk OR “high risk”)

**S6 – S4 OR S5**

S7 - (MH "Surveys+") OR (MH "Referral and Consultation+") OR (MH "Crisis Intervention") OR (MH "Early Intervention+") OR (MH "Risk Assessment")

S8 - ((“Health Surveys” OR “Screening tools” OR “screening tool” OR “Assessment” OR “assessments” OR “Referral and Consultation” OR “patient education” OR “patient educator” OR “patient educators” OR “mass screening” OR “mass screenings” OR “Crisis Intervention” OR "early medical intervention" OR ("early" AND "medical" AND "intervention") OR “risk assessment”))

**S9 – S7 OR S8**

S10 – (MH "Maternal Health Services+") OR (MH "Pregnancy+") OR (MH "Expectant Mothers")

S11 – (“Maternal Care” OR “Maternal Health” OR Perinatal OR Prenatal OR Antenatal OR Postpartum OR Pregnancy OR “Pregnant Women” OR Pregnant* OR Pregnanc* OR obstetric*)

S13 – (MH "Pregnancy Complications+") OR (MH "Abortion, Spontaneous+") OR (MH "Maternal Mortality") OR (MH "Infant, Low Birth Weight+")

S14 – (“Health outcomes” OR (health AND outcomes) OR “Pregnancy Complications” OR (pregnancy AND (complication OR complications)) OR “Fetal death” OR stillbirth OR stillborn OR “spontaneous abortion” OR “pregnancy loss” OR “pregnancy losses” OR Miscarriage* OR Miscarry OR ((medical OR health) AND (Complication OR complications)) OR "Maternal Mortality" OR (maternal AND mortalit*) OR “low birth weight” OR LBW OR “preterm birth” OR “premature birth” OR “premature labor” OR “preterm labor” OR “premature labour” OR “preterm labour”)

**S15 – S13 OR S14**

**S16 – S15 AND S12 AND S9 AND S6 AND S3** 
**N=3,369, N=828**

**Scopus** 
6/26/2019, repeated 5/6/2020

( TITLE-ABS-KEY ( "Health outcomes"  OR  ( health  AND  outcomes )  OR  "Pregnancy Complications"  OR  ( pregnancy  AND  ( complication  OR  complications ) )  OR  "Fetal death"  OR  stillbirth  OR  stillborn  OR  "spontaneous abortion"  OR  "pregnancy loss"  OR  "pregnancy losses"  OR  miscarriage*  OR  miscarry  OR  ( ( medical  OR  health )  AND  ( complication  OR  complications ) )  OR  "Maternal Mortality"  OR  ( maternal  AND  mortalit* )  OR  "low birth weight"  OR  lbw  OR  "preterm birth"  OR  "premature birth"  OR  "premature labor"  OR  "preterm labor"  OR  "premature labour"  OR  "preterm labour" ) )  AND  ( TITLE-ABS-KEY ( "Maternal Care"  OR  "Maternal Health"  OR  perinatal  OR  prenatal  OR  antenatal  OR  postpartum  OR  pregnancy  OR  "Pregnant Women"  OR  pregnant*  OR  pregnanc*  OR  obstetric* ) )  AND  ( TITLE-ABS-KEY ( barrier*  OR  bias*  OR  "delivery of health care"  OR  "delivery of care"  OR  dispar*  OR  disproportionate*  OR  "health behavior"  OR  "health knowledge attitudes practices"  OR  "health services accessibility"  OR  "health services indigenous"  OR  "indigenous health services"  OR  "health services needs and demands"  OR  inequali*  OR  inequit*  OR  "patient acceptance of health care"  OR  "patient selection"  OR  "quality of health care"  OR  "culturally competent care"  OR  "socioeconomic factors"  OR  "socioeconomically disadvantaged"  OR  "social determinants of health"  OR  underrepresent*  OR  underserve*  OR  "ethnic disparities"  OR  "ethnic disparity"  OR  "health disparities"  OR  "health disparity"  OR  "healthcare disparities"  OR  "healthcare disparities"  OR  "health-care disparities"  OR  "health care disparity"  OR  "healthcare disparity"  OR  "health-care disparity"  OR  "health status disparities"  OR  "disparities in care"  OR  "health behavior"  OR  "health behaviors"  OR  "health inequality"  OR  "health inequalities"  OR  "health inequities"  OR  "health inequity"  OR  "health related quality of life"  OR  "health-related quality of life"  OR  "health status disparities"  OR  "social class"  OR  "social disparities"  OR  "social disparity"  OR  "social factors"  OR  "social inequities"  OR  "social inequality"  OR  "social inequalities"  OR  "social inequity"  OR  at-risk  OR  "at risk"  OR  high-risk  OR  "high risk" ) )  AND  ( TITLE-ABS-KEY ( ( "Health Surveys"  OR  "Screening tools"  OR  "screening tool"  OR  "Assessment"  OR  "assessments"  OR  "Referral and Consultation"  OR  "patient education"  OR  "patient educator"  OR  "patient educators"  OR  "mass screening"  OR  "mass screenings"  OR  "Crisis Intervention"  OR  "early medical intervention"  OR  ( "early"  AND  "medical"  AND  "intervention" )  OR  "risk assessment" ) ) )  AND  ( TITLE-ABS-KEY ( "health planning"  OR  "health communication"  OR  "health services research"  OR  "delivery of health care"  OR  "delivery of care"  OR  "financial"  OR  "housing"  OR  "food insecurity"  OR  "childcare"  OR  "child care"  OR  "intimate partner violence"  OR  "safety"  OR  "social work"  OR  "social workers"  OR  "social worker"  OR  "WIC"  OR  "supplemental nutrition program"  OR  "family case management"  OR  "education status"  OR  "social welfare"  OR  employ*  OR  unemploy*  OR  "food assistance"  OR  "food supply"  OR  "hunger"  OR  "domestic violence"  OR  "social isolation"  OR  "transportation"  OR  "Psychosocial Deprivation"  OR  "Psychosocial risk" ) )

**N=4,529, N=274**

**Web of Science** 
6/26/2019, repeated 5/6/2020

#1 - TS=("health planning" OR "health communication" OR "health services research" OR "delivery of health care" OR "delivery of care" OR "financial" OR "housing" OR "food insecurity" OR "childcare" OR "child care" OR "intimate partner violence" OR "safety" OR "social work" OR "social workers" OR "social worker" OR "WIC" OR "supplemental nutrition program" OR "family case management" OR "education status" OR "social welfare" OR employ* OR unemploy* OR "food assistance" OR "food supply" OR "hunger" OR "domestic violence" OR "social isolation" OR "transportation" OR "Psychosocial Deprivation" OR "Psychosocial risk") 

#2 - TS=(barrier* OR bias* OR “delivery of health care” OR “delivery of care” OR dispar* OR disproportionate* OR “health behavior” OR “health knowledge attitudes practices” OR “health services accessibility” OR “health services indigenous” OR “indigenous health services” OR “health services needs and demands” OR inequali* OR inequit* OR “patient acceptance of health care” OR “patient selection” OR “quality of health care” OR “culturally competent care” OR “socioeconomic factors” OR “socioeconomically disadvantaged” OR “social determinants of health” OR underrepresent* OR underserve* OR “ethnic disparities” OR “ethnic disparity” OR “health disparities” OR “health disparity” OR “healthcare disparities” OR “healthcare disparities” OR “health-care disparities” OR “health care disparity” OR “healthcare disparity” OR “health-care disparity” OR “health status disparities” OR “disparities in care” OR “health behavior” OR “health behaviors” OR “health inequality” OR “health inequalities” OR “health inequities” OR “health inequity” OR “health related quality of life” OR “health-related quality of life” OR “health status disparities” OR “social class” OR “social disparities” OR “social disparity” OR “social factors” OR “social inequities” OR “social inequality” OR “social inequalities” OR “social inequity” OR at-risk OR “at risk” OR high-risk OR “high risk”)

#3 - TS=((“Health Surveys” OR “Screening tools” OR “screening tool” OR “Assessment” OR “assessments” OR “Referral and Consultation” OR “patient education” OR “patient educator” OR “patient educators” OR “mass screening” OR “mass screenings” OR “Crisis Intervention” OR "early medical intervention" OR ("early" AND "medical" AND "intervention") OR “risk assessment”))

#4 - TS= (“Maternal Care” OR “Maternal Health” OR Perinatal OR Prenatal OR Antenatal OR Postpartum OR Pregnancy OR “Pregnant Women” OR Pregnant* OR Pregnanc* OR obstetric*)

#5 - TS=(“Health outcomes” OR (health AND outcomes) OR “Pregnancy Complications” OR (pregnancy AND (complication OR complications)) OR “Fetal death” OR stillbirth OR stillborn OR “spontaneous abortion” OR “pregnancy loss” OR “pregnancy losses” OR Miscarriage* OR Miscarry OR ((medical OR health) AND (Complication OR complications)) OR "Maternal Mortality" OR (maternal AND mortalit*) OR “low birth weight” OR LBW OR “preterm birth” OR “premature birth” OR “premature labor” OR “preterm labor” OR “premature labour” OR “preterm labour”)

#6 - #1 AND #2 AND #3 AND #4 AND #5

**N=495, N=65**
